# On the Utility of Infrared Photoactivation for Native Top-Down and Complex-Down Orbitrap Mass Spectrometry of Soluble Proteoform Complexes

**DOI:** 10.1021/jasms.5c00385

**Published:** 2026-02-11

**Authors:** Cynthia Nagy, Linda B. Lieu, Christopher Mullen, Graeme C. McAlister, Rafael D. Melani, Joshua D. Hinkle, Luca Fornelli

**Affiliations:** 1 School of Biological Sciences, 6187 University of Oklahoma, Norman, Oklahoma 73019, United States; 2 Department of Chemistry and Biochemistry, 6187 University of Oklahoma, Norman, Oklahoma 73019, United States; 3 486281 Thermo Fisher Scientific, San Jose, California 95134, United States

**Keywords:** complex-down, native top-down, Orbitrap, multiproteoform complexes, photoactivation, electron transfer dissociation

## Abstract

Cellular functions arise from the coordinated action of proteoforms, which typically form multiproteoform complexes (MPCs), rather than functioning as isolated molecular entities. Deciphering the architecture and composition of MPCs is essential for linking proteoform diversity to biological function. Native top-down (nTD MS) and complex-down mass spectrometry (CxD MS) have emerged as powerful strategies to characterize MPCs, offering intact mass analysis as well as gas-phase sequencing either at the level of the complete assembly or its constituent proteoform subunits. Because the attainable sequence coverage is highly influenced by the ion activation technique, expanding activation strategies is key to improving proteoform characterization. To this end, we implemented infrared (IR) activation for the analysis of soluble MPCs—alcohol dehydrogenase (ADH; 147 kDa tetramer), enolase (96 kDa dimer), and pyruvate kinase (PK; 232 kDa tetramer). IR photons were used to induce infrared multiphoton dissociation (IRMPD) and to enhance electron-based fragmentation via activated-ion electron transfer dissociation (AI-ETD), and performance was benchmarked against higher-energy collisional dissociation (HCD). For ADH (∼36 kDa subunits), AI-ETD, HCD, and IRMPD returned similar sequence coverages in nTD MS experiments (36, 38, and 34%, respectively), with complementary cleavages resulting in a combined 48% coverage. As subunit mass increased, radical-driven fragmentation provided a clear advantage: for PK (∼57 kDa subunits), AI-ETD achieved 28% sequence coverage—approximately 15% higher than HCD or IRMPD. Together, these results highlight IR irradiation—both as a standalone dissociation modality and as a complement to electron-based activation—as a versatile strategy to enhance proteoform-level sequencing in native and complex-down MS workflows.

## Graphical abstract

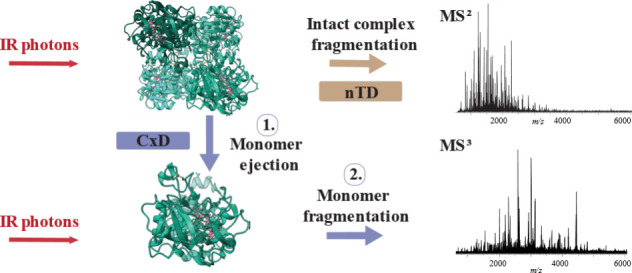

## Introduction

The remarkable complexity of biological processes is enabled by the versatility and unique physicochemical properties of biomolecules like proteins. In line with this premise, the “proteoform hypothesis” postulates that observed phenotypes are the direct product of the function of specific forms of a protein, carrying unique sets of chemical and genetic modifications, known as proteoforms.^1–3^ A growing body of evidence supports the notion that distinct proteoforms derived from the expression of the same gene can have radically different impacts on the cells and tissue in which they are present, with some maintaining homeostasis while others promoting the onset of disease.^4–6^

Mass spectrometry (MS) is the analytical technique of choice for the investigation of proteoforms through the approach termed top-down proteomics (TDP).^7,8^ Recent advances in MS and separation technologies (i.e., liquid chromatography and capillary electrophoresis) are dramatically improving the throughput of TDP analyses, enabling the identification of tens of thousands of proteoforms in a single study.^9–12^ However, TDP is typically performed under denaturing conditions, so no information regarding the quaternary structure of proteoforms can be retrieved. This aspect represents a limitation to fully unravel the biological role of proteoforms, as most of them exercise their function as part of noncovalent multiproteoform complexes (MPCs).^13^

The study of MPCs is made possible by native mass spectrometry (nMS),^14,15^ whereby native-like conditions (e.g., use of MS-compatible salts in solutions at near-neutral pH)^16^ are utilized to preserve weak, noncovalent interactions during ionization and the transfer of the analytes from the liquid to the gas phase. While the determination of the mass of an MPC is a critically important step to derive tentative stoichiometry and nature of the individual proteoform in the ensemble, gaining confident identification of such proteoforms necessitates a sequencing step.^17^ Following the nMS nomenclature proposed by Lermyte et al.,^18^ we distinguish two main approaches for the sequencing of proteoforms within MPCs. Native top-down mass spectrometry (nTD MS) entails the activation of a whole complex, which implies that multiple proteoforms may be fragmented simultaneously.^19,20^ The major benefit of nTD MS is that fragmentation spectra retain memory of the tertiary and quaternary structure of the MPC, with backbone cleavage sites typically being localized either on solvent accessible regions of individual proteoforms or in domains with particular flexibility or propensity to rapidly denature.^21^ Additionally, nTD MS experiments are relatively straightforward to perform, as they typically entail the isolation of one charge state of the MPC of interest followed by its activation. By contrast, though, the interpretation of the resulting fragmentation spectra may be complicated by their potential chimeric nature (i.e., in the case of heterocomplexes).

Differently, the second approach, known as complex-down MS (CxD MS), relies on a multistage ion activation workflow culminating in the characterization of individual monomers released from an MPC.^22,23^ Specifically, relatively mild activation is first utilized to eject one or multiple subunits, either after isolating one charge state of the whole MPC and increasing its internal energy (i.e., by performing a tandem MS experiment, or MS^2^) or by increasing the energy of in-source collisional dissociation (sCID), generating a pseudo-MS^2^ mass spectrum. After this step, which is also referred to as complex-up MS, liberated monomers (i.e., individual proteoforms) can be further isolated for subsequent activation and gas-phase sequencing, through the acquisition of an MS^3^ or pseudo-MS^3^ mass spectrum. The general consensus postulates that ejected monomers are denatured, a notion supported by both empirical evidence—for instance, the phenomenon known as asymmetric charge partitioning^24–26^ —and computational simulations.^27,28^ This implies that, while the CxD MS approach allows for the isolation and fragmentation of individual proteoforms, avoiding the generation of chimeric mass spectra, the observed fragmentation patterns do not reflect structural features of monomers in their native-like conformation. Overall, nTDMS and CxD MS represent complementary tools for the interrogations of MPCs, the former being important for structural biology and allowing for the determination of fine structural features such as the exact location of ligands,^29,30^ with the latter facilitating the untargeted investigation of MPCs within cells and tissues.^31,32^

In this context, the choice of ion activation has profound impacts on experimental results. Collision-based activation, including resonant collision-induced dissociation (CID) and beam-style collisional activation, also known as higher-energy collisional dissociation (HCD) in Orbitrap-based instruments, is particularly effective for both stripping a monomer from an MPC in CxD MS experiments, and fragmenting the monomer backbone in nTD MS as well as CxD MS. Interestingly, a recent report suggests that collision-induced dissociation can probe higher-order structure of MPC subunits.^33^

Pioneered by Wysocki and co-workers, surface-induced dissociation (SID) is highly effective in dissociating MPC subunits by inducing only minimal changes in their secondary/tertiary structure.^34,35^ Therefore, SID represents a formidable structural biology tool, particularly when coupled with ion mobility MS.^36^ For this same reason, though, SID is less suitable for obtaining sequencing information on MPC monomers in nTD MS and CxD MS studies.

Ultraviolet photodissociation (UVPD) can also be applied to induce the dissociation of MPCs into individual subunits, with a degree of monomer denaturation that can be modulated by adjusting the UV laser pulse energy.^37^ Additionally, UVPD has been proven effective in characterizing monomers derived from both soluble and membrane MPCs through nTD MS and CxD MS experiments.^38–40^

Radical-driven fragmentation methods, particularly electron capture dissociation (ECD) and electron transfer dissociation (ETD), have been extensively leveraged for extending the sequence coverage of MPC subunits and pinpointing the location of post-translational modifications and ligand-binding sites.^41–43^ However, due to their propensity to preserve noncovalent interactions, they are generally inefficient in provoking MPC disassembly. ECD and ETD primarily induce backbone cleavages within solvent-accessible regions and can be applied to either the whole MPC or ejected subunits.^42,44–46^ In contrast, electron–ionization dissociation (EID)—an approach largely untapped in the native MS domain—has been shown to yield rich sequence information, complementary to that of ECD, in part due to its ability to disrupt subunit interaction interfaces.^47^ Importantly, instruments equipped for ECD can also generate the high-energy electrons (>20 eV) required for EID, which may position this technique as a readily accessible and promising approach for native MS workflows.

Finally, infrared (IR) irradiation has historically played an important role in the native MS of MPCs. Utilized as a slow-heating ion dissociation method—infrared multiphoton dissociation (IRMPD)—it has shown performance in line with resonant CID, both with regard to monomer ejection and sequencing.^19,48^ As a means to provide supplemental activation for enhancing the sequencing performance of ECD—in activated-ion ECD (AI-ECD) experiments—IR irradiation compensated for the modest sequencing performance that ECD demonstrated for specific MPCs, likely due to difficulties in releasing product ions after their generation due to residual noncovalent interactions.^49^

Here we present a first attempt to benchmark the potential of infrared photoactivation for the nTD MS and CxD MS analysis of soluble MPCs using a modified Tribrid Orbitrap mass spectrometer equipped with a CO_2_ laser. Recently, a similar instrument setup has been utilized for the native MS analysis of membrane proteins and membrane protein complexes, whereby IR photon irradiation was proven superior to conventional in-source CID or even beam-style collisional dissociation for the removal of residual lipids and detergent adducts.^50–52^ In the present study, we specifically focus on the fragmentation performance of IRMPD and activated-ion ETD (AI-ETD)^53^ in comparison to HCD, investigating MPCs with monomers spanning from 36 to 57 kDa in mass. While preliminary and based only on the analysis of three standard MPCs, our results not only demonstrate the complementarity of the three evaluated ion dissociation methods but also suggest that they could find optimal use in different applications of native MS, from structural biology to high-throughput MPC characterization.

## Experimental Section

### Sample Preparation

All standard proteins—enolase from *Saccharomyces cerevisiae* (Part #E6126-500UN), pyruvate kinase (PK) from rabbit muscle (Part #P1506-5KU) and alcohol dehydrogenase (ADH) from *S. cerevisiae* (Part #A8656)—were obtained from Millipore Sigma (St. Louis, MO). Samples were solvent-exchanged into 150 mM ammonium acetate solution (pH 6.9) using 30 kDa molecular weight cutoff Amicon centrifugal filter units (Sigma) as described previously.^23^ Final sample concentration was 7 μM in all cases (with respect to the complex).

### MS Instrumentation

MS measurements were performed on a modified Orbitrap Ascend Tribrid mass spectrometer (Thermo Scientific, San Jose, CA) equipped with a 10.6 μm CO_2_ laser (30 W, C-30, Coherent) directed into the high pressure linear ion trap (LIT). Laser powers used in this study will be indicated as nominal wattage values. Samples were directly infused with Au-coated borosilicate emitters using a NanoFlex electrospray source (Thermo Scientific). The instrument was operated in high-pressure mode (15 mTorr in front and back ion-routing multipoles [IRM]). Source parameters were typically set at the following values: 1100–1300 V electrospray voltage, 300 °C transfer tube temperature, 30% RF lens, 75–250 V source collision-induced dissociation (sCID) and sCID compensation scaling factor of 0.01–0.04. Broadband (MS^1^) spectra were collected in the Orbitrap mass analyzer at resolving power (r.p.) of 7500, 15,000 and 30,000 (at *m*/*z* 200). Monomers were ejected either by increasing sCID energy in the source region (pseudo MS^2^ or pMS^2^) or in the front IRM using higher-energy collisional dissociation (HCD) (MS^2^). Fragmentation of the whole complexes and the monomers was carried out using HCD, infrared multiphoton dissociation (IRMPD), electron-transfer dissociation (ETD) with and without concurrent photoactivation—the former being called activated-ion ETD (AI-ETD)—and ETD with supplemental HCD activation (EThcD). Fragmentation spectra were recorded in the Orbitrap at 240,000 r.p. (at 200 *m*/*z*), with automatic gain control (AGC) target value set at 2000% with a maximum injection time of 3000 ms, averaging 150 time-domain transients.

### Data Analysis

Deconvolution of intact mass spectra (available on MassIVE under repository number MSV000099804) and the manual validation of assigned product ions were carried out using ProSight Native (Proteinaceous Inc.; Evanston, IL),^54^ which integrates TDValidator.^55^ Validation was performed using the following values: signal-to-noise ratio (S/N) threshold for peak picking: 10; fragment mass tolerance: 10 ppm; interisotopic tolerance: 3 ppm; minimum score threshold: 0.50. Spectra were off-line recalibrated (detailed in the Supporting Information, Figure S1). The following ion types were considered for matching: *b*- and *y*-ions for HCD and IRMPD; *c*- and *z*-ions for ETD; *b*-, *c*-, *y*- and *z*-ions for AI-ETD and EThcD.

Manually validated files were subsequently imported into the Structural Viewer module of ProSight Native for 3D visualization of the detected fragment ions. For alcohol dehydrogenase (ADH), the 7KCB protein structure was obtained from the RCSB Protein Data Bank and modified in ChimeraX 1.10.1 using the swappaa command to generate the predominant proteoform observed (substituting the native residues, residue 58: T and 151: V).^56^ The 3D structures for enolase and pyruvate kinase (PK) were obtained using UniProt accession codes P00924 and P11974, respectively.

Product ion abundance (PIA) plots were generated using an in-house Python script. Briefly, fragment ion intensities were extracted from the manually validated TDValidator output Excel files and normalized by their respective charge states. When multiple fragments shared the same annotation, their charge-normalized intensities were summed. All resulting values were then normalized to the most intense product ion and expressed as percentages for visualization. Fragment positions were assigned based on their nomenclature: for *b*- and *c*-type ions, the original positional numbering was retained, whereas for *y*- and *z*-type ions, positions were referenced to the N-terminus.

## Results and Discussion

### Infrared Irradiation as a Versatile Tool for the Analysis of Multiproteoform Complexes

All three selected MPCs (alcohol dehydrogenase tetramer; enolase dimer; pyruvate kinase tetramer) were characterized using both nTD MS and CxD MS and probed by three different ion activation techniques—namely, HCD, IRMPD and AI-ETD (Figure 1 and Figure S2). MS^1^ analyses suggested the likely composition of MPCs, including proteoform subunits and associated cofactors/ligands (Table S1). For ADH, the dominant species corresponded to the homotetramer carrying eight Zn^2+^ ions (two per subunit). The subunits had the initiator methionine cleaved (indicated as MetOFF) and exhibited two sequence variations relative to the canonical UniProt entry (P00330)—namely, I151 V and V58T,^23,33,43^ the presence of the latter being confirmed by several product ions (Figure S3). In the nTD MS approach, a single charge state of the tetramer (25+) was isolated in the quadrupole and interrogated with HCD, IRMPD and AI-ETD. In contrast, the CxD MS workflow included (i) monomer ejection (via true MS^2^ or pMS^2^), (ii) isolation of a single charge state (17+) of the released ADH monomer, and (iii) gas-phase sequencing of the selected monomer with the same dissociation methods applied in nTD MS.

**Figure 1:**
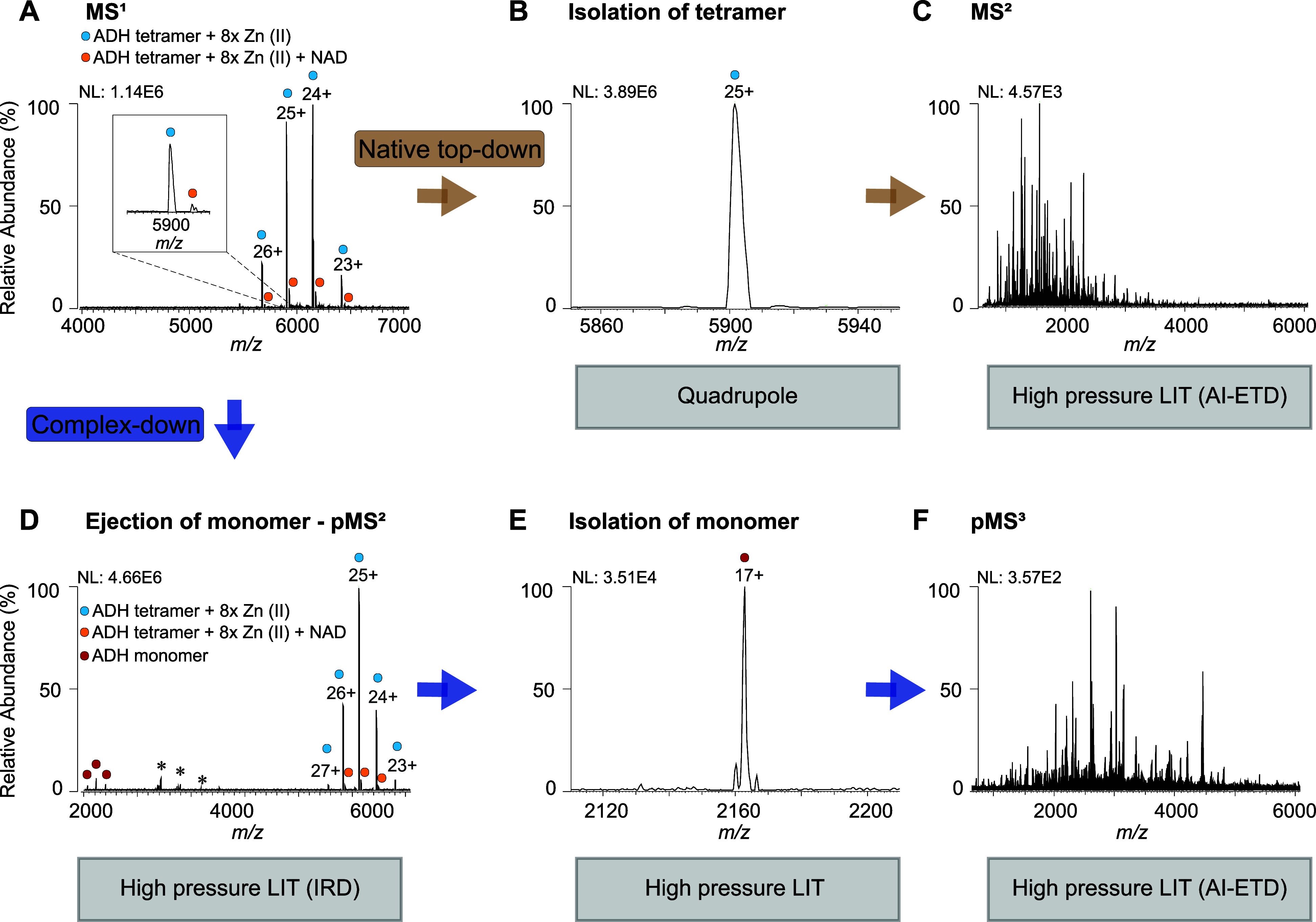
Workflow for nTD MS and CxD MS represented through the AI-ETD measurements of ADH. (A) Intact mass measurement. (B) In nTD, a single charge state of the intact complex is isolated (isolation width: 25 *m*/*z*) and (C) fragmented. (D) For CxD MS, monomers are ejected from the complex (here using IRD in the high pressure LIT), and (E) a single charge state of the monomer is isolated (isolation width: 25 *m*/*z*) and (F) subjected to gas-phase dissociation. Gray boxes below the panels indicate where that specific event is performed. Asterisk (*) in panel (D) denotes an impurity.

Complex-up analysis (i.e., intact mass measurement of ejected subunits) uncovered an additional ADH monomer proteoform exhibiting −44 Da mass difference relative to the dominant species, in agreement with earlier observations for yeast ADH.^23,43^ Intriguingly, Li et al. observed −44 Da shifts exclusively for *c*-type ions,^43^ which is suggestive of the modification residing near the N-terminus. This is (partly) consistent with the present findings: a −42 Da mass shift relative to the major subunit proteoform (MetOFF, N-term acetylation, V58T, I151 V) was observed for *b*-type ions in IRMPD and HCD nTD MS experiments, indicating that a portion of the mass deficit may correspond to the absence of N-terminal acetylation (NtAc) (Figure S4). However, the lack of NtAc alone does not fully account for the observed mass difference, which implies the presence of an additional sequence variation—potentially a V→T substitution—likely positioned toward the central region of the sequence. It is also important to note that the proteoform lacking NtAc was present at a substantially lower abundance, leading to low-intensity diagnostic ions with reduced S/N, whose isotopic distributions often showed partial overlap with those of other fragments. Confident assignment was nonetheless possible due to the high r.p. employed for collecting fragmentation spectra in the Orbitrap mass analyzer (240,000 at *m*/*z* 200).

As shown in Figure 1, a single ion activation technique (in this case AI-ETD) can generate markedly different fragmentation patterns in nTD and CxD MS. This behavior likely reflects differences in site accessibility, as the compact tetrameric assembly imposes greater structural constraints than the liberated subunit (which is less native-like as suggested by asymmetric charge partitioning). Accordingly, the tetramer and the monomer exhibit largely different charge densities (0.17 and 0.47 number of charges/1000 Da, respectively). These distinct charge densities further modulate fragmentation behavior, with ETD having the well-established preference for highly charged ions and HCD favoring intermediate to lower charge states.^57,58^ Despite ETD being largely charge-dependent, concurrent IR irradiation has been shown to significantly enhance ETD product ion yield across a broad charge-state envelope. Notably, the benefit of IR photoactivation for lower charge states becomes increasingly pronounced with increasing molecular weight (Mw). For example, in the case of denatured carbonic anhydrase (∼29 kDa), AI-ETD doubled the number of unique fragments moving from a charge density of 0.93 to 0.69.^58^ This feature of AI-ETD can be attributed both to the dissociation of ETnoD species and to the partial unfolding of polypeptide chains, which can prove particularly favorable for the sequencing of native assemblies.

Efficient desolvation and declustering are essential in native MS and native top-down workflows, as residual solvent and buffer adducts broaden spectral features, decrease mass accuracy, and compromise fragmentation efficiency. Historically, in-source collision-induced dissociation (sCID) has served as the primary method for promoting solvent and adduct removal. In this approach, all ions experience elevated acceleration voltage early in the ion path, leading to energetic collisions that can effectively reduce nonspecific adduction. However, these collisions may perturb fragile noncovalent interaction and can induce premature subunit ejection. For membrane-protein complexes, sCID is often limited in its ability to efficiently remove detergent micelles, resulting in heterogeneous mass spectra in which the proteins remain (partially) associated with the detergent. In contrast, IR photoactivation has been shown to liberate intact membrane-protein assemblies from their detergent environment while preserving complex integrity.^50,52,59^ The more efficient removal of micelles in the case of IRD can been attributed to their IR photon absorption properties.^52^ Detergents exhibit IR-active vibrational modes whose absorption bands overlap with the wavelength of the CO_2_ laser (typically 10.6 μm), which is believed to cause the preferential activation and disruption of the micellar layer. While the importance of IR activation for membrane-protein complexes is now well-established, its utility is not restricted to micelle disruption. IR irradiation can also promote the desolvation and declustering of soluble protein assemblies,^60–65^ a capability that—despite its longer history—has received comparatively less attention. Nevertheless, it holds significant value for nMS and nTD analysis. Notably, Freitas et al. demonstrated the in-trap cleanup of a 59 kDa oligopeptide binding protein, where S/N was increased by an order of magnitude through the IR irradiation of trapped ion populations.^62^ More recently, Hale et al. showcased the power of IR-assisted declustering on a prototype IR-LIT instrument for the analysis of MPCs directly from tissue using nanospray-desorption electrospray ionization. IR declustering was found to outperform collisional activation in decreasing the chemical background arising from nonspecific clusters.^64^ To investigate this potential, we directly compared IR-assisted desolvation (IRD) with sCID for ADH tetramer (Figure S5). Although peak widths were on par, IR induced a slight decrease in the observed *m*/*z* values for individual tetramer charge states, indicative of more effective solvent/adduct removal. Correspondingly, deconvolution of IRD spectra returned experimental mass values closer to the theoretical ones, further supporting the desolvation efficiency of IR activation. More specifically, the Mw difference observed between spectra obtained with IRD and sCID (with maximum potential, 250 V) is ∼13 Da. Essentially, IRD yielded more accurate Mw information than sCID for two of the three standard MPCs. The exception was PK, which—presumably due to its high Mw (∼232 kDa)—benefited from using voltage rollercoaster filtering (VRF).^66^ VRF allows improved transmission of large Mw species via altering the voltage scheme of the front-end optics, where sCID plays an important role in determining the balance between declustering and the kinetic energy management of complexes.

In addition to promoting desolvation, IR activation can also be used to dissociate MPCs and release monomer subunits. Prior work has shown that IR-driven dissociation can display either SID- or CID-like characteristics depending on the irradiation regime (pulsed vs continuous IR radiation).^59^ The continuous-wave CO_2_ laser used in our experiments yielded monomers whose charge densities were comparable to those observed for sCID and HCD. While increasing HCD NCE% produced a progressive shift toward higher charge states (consistent with structural perturbation and unfolding), raising the laser power did not appreciably alter the monomer charge state distribution for the range of laser powers tested (Figure S6).

### Similarities and Differences of Monomer Fragmentation via HCD and IRMPD

HCD was utilized as the ion dissociation method of reference to obtain sequence information on MPCs’ subunits due to its high fragmentation efficiency (defined as the capability of converting precursor ion current into sequence-informative product ion current) and its widespread use in nMS studies, particularly those based on hybrid quadrupole-Orbitrap instruments.^23^ Additionally, as a vibrational energy threshold ion activation method, the HCD mechanism allows for a direct comparison with IRMPD, which represents the first IR-based fragmentation method enabled on the modified Tribrid Orbitrap mass spectrometer benchmarked in the present study. In the case of all investigated MPCs, HCD outperformed IRMPD in terms of sequence coverage, both when applied in CxD MS and in nTD MS experiments (Figure 2 and Figures S7 and S8). Counterintuitively, the sequence coverage values obtained for the MPCs’ subunits in nTD MS experiments were higher than those from CxD MS experiments (Table S2). The unfolding process that accompanies monomer ejection in CxD MS should enable—as demonstrated by the present data—the fragmentation of sequence regions inaccessible when these proteoforms are involved in subunit–subunit interactions (as it is the case for nTD MS). We attribute the reduced sequence coverage reported for CxD MS experiments to the substantially lower intensity (on average, 1 order of magnitude) of product ion signals detected from ejected subunits (as a consequence of the low signal of the precursor ion). With regard to ADH, IRMPD sequence coverage (27% and 34% for CxD MS and nTD MS, respectively) exceeds what is reported in the literature for vibrational energy threshold methods; typically, collision-based activation yields sequence coverage between 3% and 20%.^67^ In light of this, the HCD results reported here (sequence coverage of 35% and 38% for CxD MS and nTD MS, respectively) appear even more surprising. Notably, in the Orbitrap Ascend Tribrid mass spectrometer the HCD fragmentation of quadrupole-isolated precursors (MS^2^ experiments for nTD MS and pMS^3^ experiments for CxD MS) is performed in the front ion-routing multiple (fIRM), unlike in previous series of Tribrid instruments and in hybrid quadrupole-Orbitrap systems, where the back IRM (i.e., positioned after the C-trap along the ion path) is used. While the exact advantages of performing HCD in the fIRM should be further investigated in the context of nMS, this may partially explain the unexpected efficacy of HCD in sequencing MPCs’ subunits.

**Figure 2:**
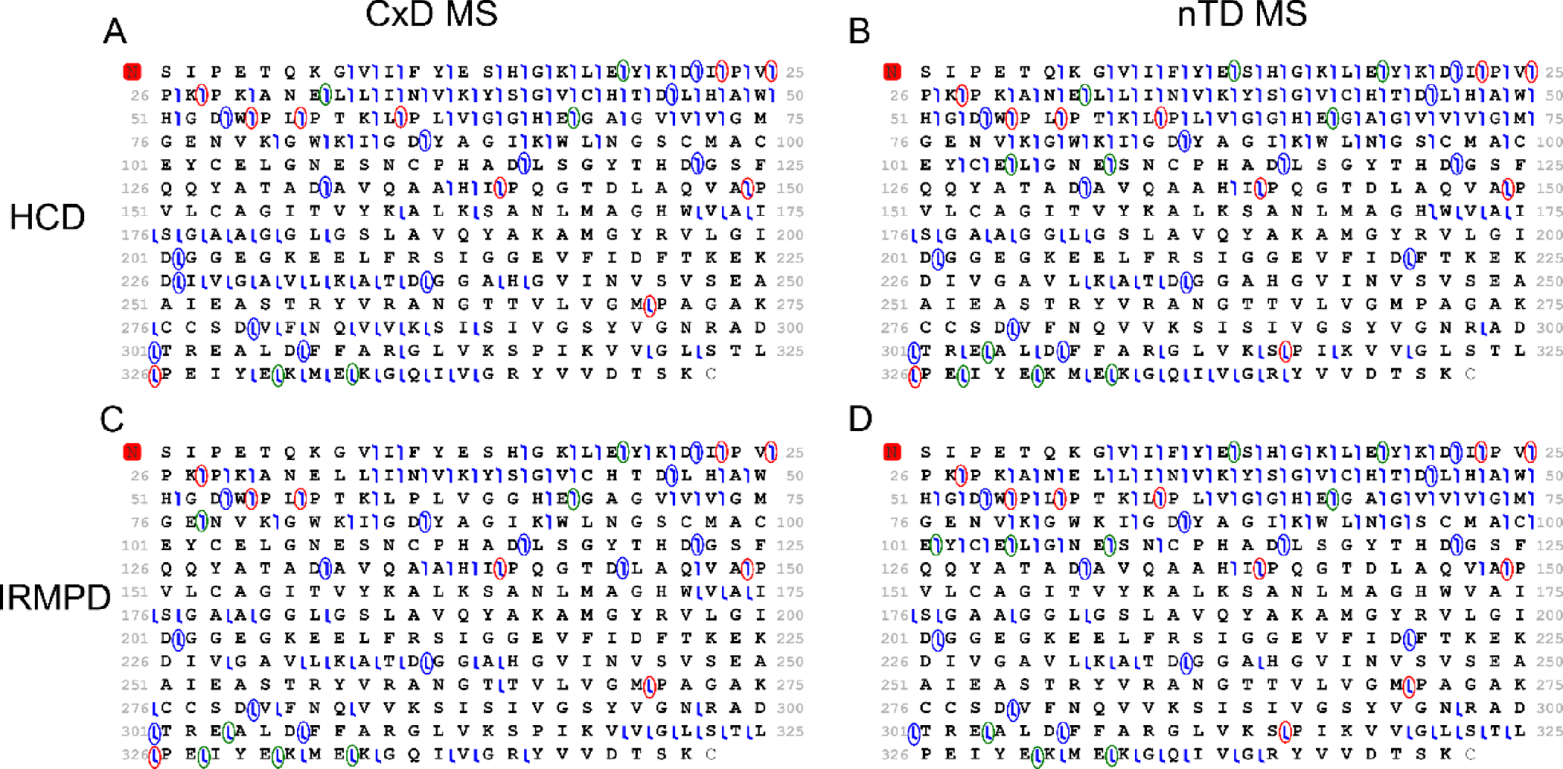
Fragmentation maps of the ADH monomer obtained via HCD (A) in CxD MS and (B) nTD MS experiments. Fragmentation maps of the ADH monomer obtained via IRMPD (C) in CxD MS and (D) nTD MS experiments. Red circles mark fragments occurring N-terminal to proline residues; blue and green circles mark fragments occurring C-terminal to aspartic acid and glutamic acid residues, respectively. N-terminus highlighted in red signified N-terminal acetylation. HCD NCE (A) 60% and (B) 50% and IRMPD laser power (C) ∼9 W and (D) ∼6.9 W.

In agreement with that reported by Ives et al.,^68^ both HCD and IRMPD demonstrated clear propensities for cleaving the backbone at hotspots. Hotspots reported previously for HCD—N-terminal side of Pro (red circles in Figure 2) and C-terminal side of Asp (blue circles)—are accompanied here by the cleavage at the C-terminal side of Glu (green circles) both in the case of HCD and IRMPD.

However, this qualitative analysis does not show clear differences between IRMPD and HCD, but rather different distributions of sequenced regions in CxD MS and nTD MS experiments (*vide infra*). On the contrary, the quantitative evaluation of fragmentation products, or product ion abundance (PIA) analysis,^69,70^ highlights unique features of these two ion activation methods (Figure 3). HCD product ions forming a sequence tag demonstrate in most cases similar abundance levels. Interestingly, this is true whether consecutive fragments are localized around a fragmentation hotspot (i.e., cluster at residues 225–238 from CxD MS) or not (i.e., cluster at residues 170–180). Conversely, IRMPD shows a much stronger tendency to cleave at fewer hotspots, as evidenced by the normalized PIA plots, which display fewer but more intense signals, while the remaining backbone cleavages show particularly low intensities (often <10% of the base peak). In line with what was proposed by Ives et al. for HCD,^68^ we believe this feature of IRMPD, also observed for enolase and pyruvate kinase (Figures S9 and S10), could be leveraged to construct a native fragmentation propensity score. We posit that the tendency of IRMPD to yield highly predictable backbone cleavage of MPCs’ subunits could be advantageous for applications of native top-down MS performed on the separation time scale, where there is limited possibility of averaging time-domain transients or mass spectra.^71^

**Figure 3:**
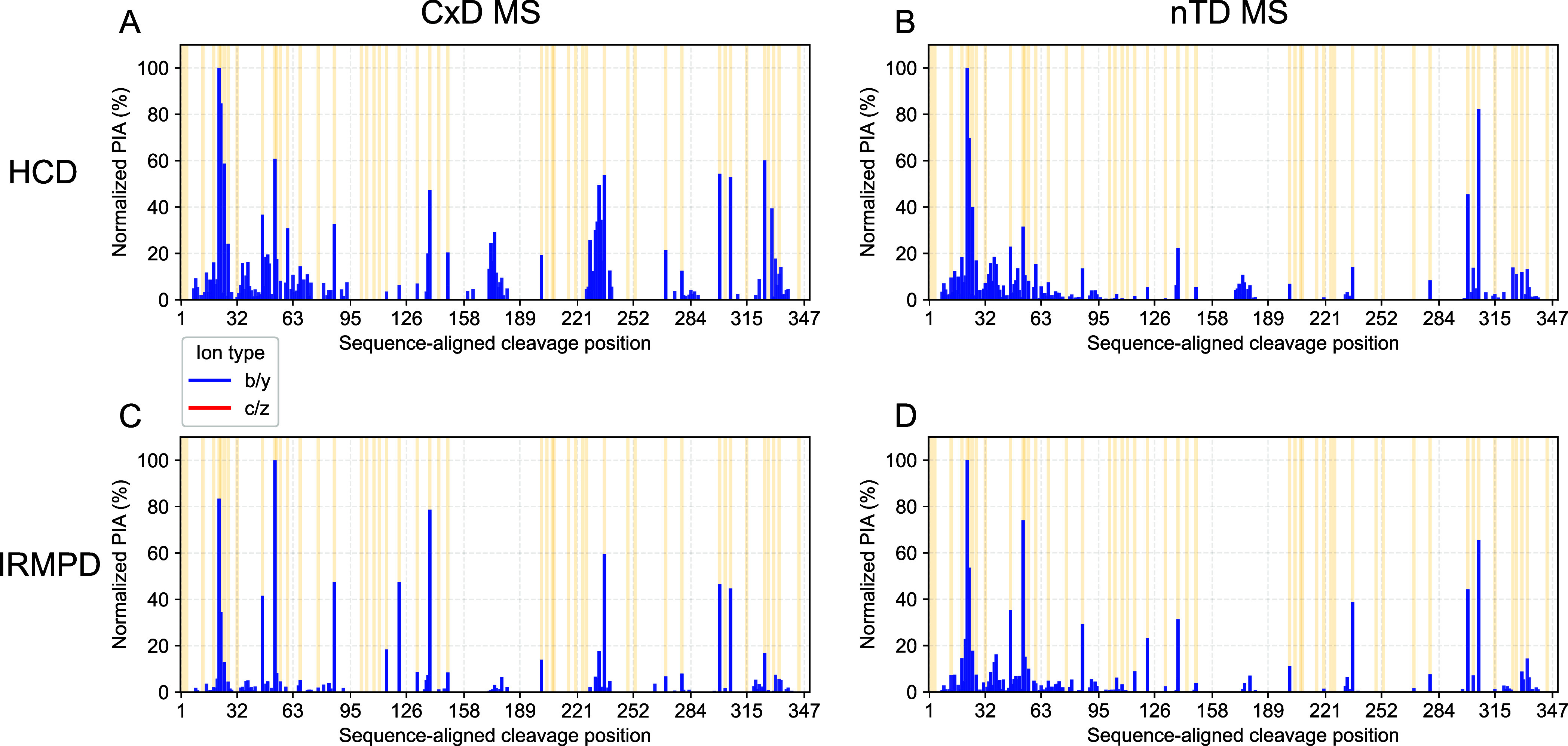
Results of product ion abundance (PIA) analysis for HCD and IRMPD experiments on ADH, comparing CxD MS and nTD MS. (A) HCD CxD MS, (B) HCD nTD MS, (C) IRMPD CxD MS, and (D) IRMPD nTD MS. Regions highlighted in yellow indicate D/*, E/*, and */P cleavage sites. HCD NCE (A) 60% and (B) 50% and IRMPD laser power (C) ∼9 W and (D) ∼6.9 W.

### AI-ETD Fragmentation Pattern Complements IRMPD and HCD

Radical-driven fragmentation methods like ETD are considered orthogonal to both slow-heating methods and ultraviolet photodissociation, as demonstrated by several top-down MS reports.^55,72^ Therefore, AI-ETD, the second ion dissociation method enabled by IR irradiation on the modified Orbitrap Ascend to be evaluated in this study, was tested both in CxD MS and nTD MS scenarios. First, AI-ETD was benchmarked against ETD and EThcD using ADH. Both when dissociating directly the ADH tetramer and the ejected monomer, AI-ETD outperformed the related electron-based methods (Table S3), rivaling HCD in terms of sequencing efficiency in the case of nTD MS (36% versus 38% sequence coverage for AI-ETD and HCD, respectively). For both CxD MS and nTD MS, optimal fragmentation parameters (i.e., those resulting in the highest sequence coverage) for AI-ETD resulted in a significantly larger fraction of *b*/*y*-type ions compared to EThcD (Table S3). This suggests that for both a native-like and a partially denatured monomer, optimal results in AI-ETD are achieved by tuning the laser power to induce mild IRMPD, rather than simply unfolding the precursor ion during the ETD reaction. Based on the fact that nTD MS is the scenario in which AI-ETD produces the largest performance gap against ETD and EThcD (sequence coverage of 35.5%, 14.2% and 11.8% for AI-ETD, EThcD and ETD, respectively), we hypothesize that ETD alone cannot efficiently fragment the solvent-inaccessible core of the ADH subunits when these are folded in their native-like conformation.^73^ We further hypothesize that HCD postactivation of ETD products (i.e., EThcD) is globally not as effective as IR irradiation-induced unfolding occurring simultaneously to ETD in yielding sequence information. Additionally, in the EThcD nTD experiments of PK, we began to observe the emergence of intense signals when the supplemental activation exceeded 50% NCE. Under these elevated activation conditions, a substantial portion of the ion current remained unassigned when only terminal c/z and b/y ions were considered (data not shown). Enabling the internal fragment matching option in TDValidator allowed these signals to be matched; however, the high degree of ambiguity precluded confident assignment.

AI-ETD displays a substantial level of complementarity in terms of generated backbone cleavages compared to both HCD and IRMPD, particularly in the case of nTD MS experiments. The number of unique backbone cleavages yielded by AI-ETD is significant for ADH and becomes progressively larger with the mass of the MPCs’ subunits (Figure 4 and Figures S7 and S8). Particularly, in the case of the 57 kDa PK subunit, AI-ETD returns the largest fraction of unique backbone cleavages (75 and 88 unique cleavages for CxD MS and nTD MS, respectively, versus 10 and 16 observed for HCD). In addition, AI-ETD of ADH tetramer generated the highest number of N-terminal fragments still carrying a Zn­(II) ion (Figures S11 and S12).

**Figure 4:**
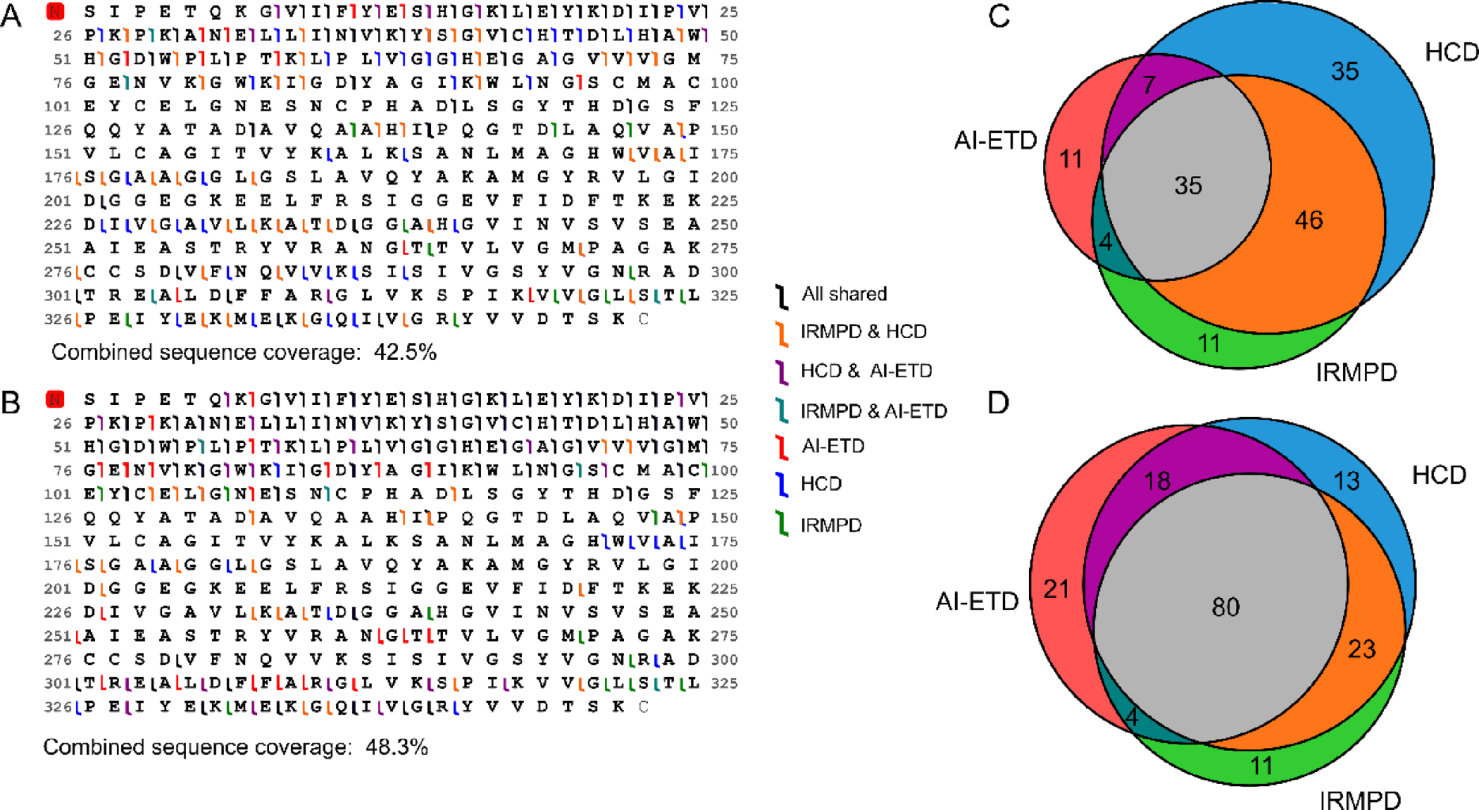
Comparison of unique product ions in AI-ETD, HCD, and IRMPD experiments on ADH at the (A) CxD MS and (B) nTD MS levels, shown as fragmentation maps. Venn diagrams display the distribution of unique product ions at the (C) CxD MS and (D) nTD MS levels. Given the different types of product ions produced by AI-ETD and IRMPD/HCD, Venn diagrams are based on backbone cleavage regardless of the nature of matched product ions. CxD experiments: HCD NCE 60%, IRMPD laser power ∼9 W, AI-ETD with ∼4.5 W laser power and 15 ms ETD reaction time; nTD experiments: HCD NCE 50%, IRMPD laser power ∼6.9 W, AI-ETD with ∼2.7 W laser power and 10 ms ETD reaction time.

Finally, while vibrational energy threshold methods were competitive or even outperformed AI-ETD in terms of achieved sequence coverage for the MPCs with smaller subunits (i.e., 36 kDa ADH and 46 kDa enolase), in the case of the 57 kDa PK, AI-ETD returned by far the most extensive sequencing (23.3% and 27.8% for CxD MS and nTD MS, respectively), essentially doubling the sequence coverage values yielded by IRMPD and HCD (Table S1). These results are in line with a recent work in which AI-ETD was used for the fragmentation of 66 kDa human serum albumin, where the precursor ion charge density was not particularly high even under denaturing ionization conditions due to extensive presence of disulfide bonds that limited protonation.^74^

### Sequencing Patterns in nTDMS Provide Structural Information on the Original MPC

The two approaches used in this study interrogate MPCs at different structural levels: as mentioned before, CxD MS targets the released subunit, while in nTD MS the intact complex is fragmented—a distinction that suggests the two workflows should provide complementary sequence information, as they probe different structural states. Because nTD MS activates the intact assembly, it can preserve elements of quaternary organization at the moment of cleavage and thus report on solvent exposure and interfacial protection within the native complex. By contrast, CxD MS introduces an intermediate ejection step (generally via vibrational activation) that perturbs subunit interfaces and can promote partial unfolding of the monomer. This distinction is reflected in our PIA plots for ADH in Figure 3. Using HCD and IRMPD, nTD MS emphasizes regions consistent with solvent exposure of the MPC, whereas CxD MS provides increased access to central and C-terminal segments that are otherwise shielded within the native assembly. This observation shows that vibrational activation—despite its established role in promoting complex unfolding—can retain quaternary-structure information, which is in line with that reported by Lantz et al.^33^ Radical-driven fragmentation methods (ECD, ETD) can offer an even clearer readout of higher-order organization, as they cleave backbone bonds preferentially over noncovalent interactions. Consistent with prior observations for the nTD MS analysis of ADH,^19,42,44–46,73^ this behavior is also reflected in the AI-ETD PIA plot obtained in the present study (Figure 5). In nTD MS mode, AI-ETD generates an extensive series of *c*-type ions extending to residue N110, with only minor contributions from *z* (or *y*) -ions. The deep N-terminal coverage obtained herein can be attributed to concomitant IR irradiation during AI-ETD, which promotes partial unfolding that extends toward the core, without fully disrupting the native quaternary architecture. This controlled-activation regime is similar to that described by Zhang et al., wherein stepwise increases in internal energy through source acceleration voltage promoted progressive unfolding of ADH.^42^

**Figure 5:**
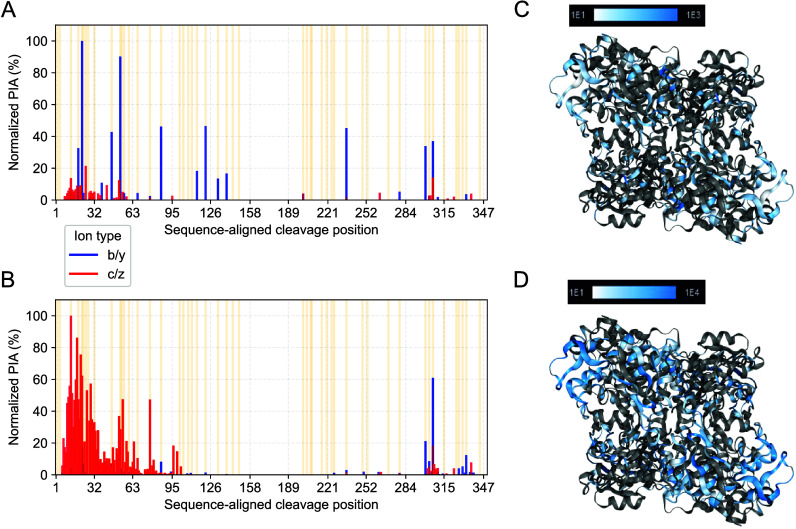
Product ion abundance (PIA) plots for AI-ETD fragmentation of the ADH (A) in CxD MS and (B) nTD MS experiments. Regions highlighted in yellow indicate D/*, E/*, and */P cleavage sites, while *b*/*y*-ions are displayed in blue and *c*/*z*-ions in red. (C, D) Crystal structure of ADH with assigned product ions from CxD and nTD MS experiments, respectively. Matched backbone cleavages are colored in shades of blue as a function of their intensity. (A) AI-ETD with ∼4.5 W laser power and 15 ms ETD reaction time; (B) AI-ETD with ∼2.7 W laser power and 10 ms ETD reaction time.

The CxD AI-ETD PIA plot exhibits a markedly different profile. To obtain appreciable sequencing following subunit ejection, substantially higher IR laser power was required, and the resulting spectra were dominated by *b/y*-type ions, with only minor contributions from *c/z-*ions. This indicates that for the CxD MS analysis of ADH, efficient fragmentation arises primarily from IR-driven vibrational activation rather than electron-based backbone cleavage, in striking contrast to the behavior observed for nTD MS. To our knowledge, this requirement for strong IR assistance and the resulting *b/y*-dominated fragmentation in CxD AI-ETD has not been previously reported. Importantly, AI-ETD in CxD mode still yielded information from central regions of the ADH sequence that remain largely inaccessible in nTD MS, consistent with partial unfolding of the monomer upon release (as could be seen in Figure 3 as well, for HCD and IRMPD). Interestingly, even these cleavages within the central portion of the sequence were dominated by *b/y*-ions, suggesting that electron transfer alone is insufficient to generate or liberate larger fragments in the ejected monomer. Comparable behavior was observed for enolase and PK (Figures S13 and S14), though each complex displayed distinct fragmentation signatures reflecting differences in quaternary structure.

Our previous nTD MS work on a monoclonal antibody demonstrated that combining multiple, orthogonal fragmentation strategies increases sequence coverage.^75^ Integrating the results from all three activation methods tested here (AI-ETD, HCD, and IRMPD) in nTD MS experiments yielded 48.3%, 43.2%, and 32.7% sequence coverages for ADH, enolase, and PK respectively. These values could be elevated further by combining the results with those of CxD, returning coverages of 55.8% (ADH), 45.1% (enolase), and 46.5% (PK) (Figure 6 and Figures S15 and S16). The magnitude of improvement depended on the quaternary structure of each MPC. Enolase exhibited only a modest gain, consistent with both termini being solvent-exposed in the native complex. In contrast, PK showed a substantial increase (∼14% higher coverage), in agreement with its crystal structure, where both N- and C-termini participate in subunit interfaces.

**Figure 6:**
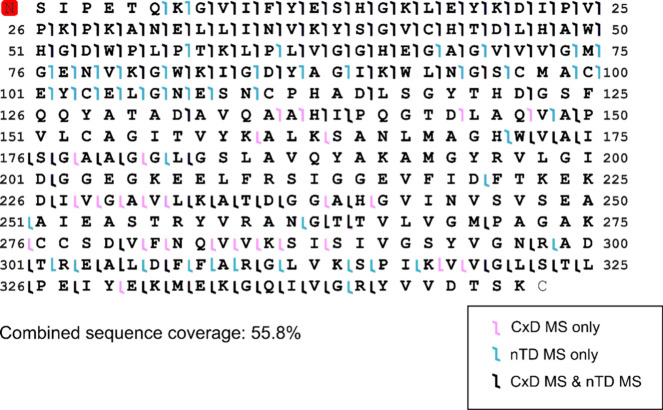
Combined fragment map showing results from AI-ETD, HCD, and IRMPD experiments for ADH analyzed via CxD MS and nTD MS. Pink brackets indicate fragments detected only in CxD MS experiments, blue brackets indicate fragments detected only in nTD MS experiments, and black brackets indicate fragments detected in both. CxD experiments: HCD NCE 60%, IRMPD laser power ∼9 W, AI-ETD with ∼4.5 W laser power and 15 ms ETD reaction time; nTD experiments: HCD NCE 50%, IRMPD laser power ∼6.9 W, AI-ETD with ∼2.7 W laser power and 10 ms ETD reaction time.

Although these gains align with the intuitive expectation that monomer release should expose buried regions and facilitate deeper sequencing, our results suggest that the extent of this “unmasking” may be more limited than typically assumed. Traditionally, the disproportionately high charge carried by the released monomer has been rationalized by charge migration to an unfolding subunit.^25,26^ This concept is substantiated by the collision-induced unfolding of MPCs monitored by ion-mobility MS (IM-MS). Changes in collisional cross-section (CCS) point to different conformational states, where an expanded structure can be attributed to the unraveling of the protein backbone.^76,77^ After ejection, the monomer is expected to take on a more elongated structure, one that can accommodate the relatively high number of charges. Such an extended structure would be expected to exhibit enhanced susceptibility to fragmentation and to yield a broader array of fragment ions than its folded counterpart. However, our experiments show that while the sequence coverage is not higher (in absolute terms) for the ejected monomer, CxD enabled the interrogation of regions of the polypeptide chain that remained unexplored by nTD. This observation implies that monomer release is accompanied by structural rearrangement, rather than a simple uniform unfolding of the monomer. This interpretation is in agreement with the behavior reported by Breuker and co-workers, who demonstrated that gas-phase unfolding of ubiquitin and cytochrome c ions proceeds through an ensemble of intermediates, and is strongly influenced by the localization of charged sites instead of charge state alone.^78,79^ In these studies, the ECD yield of gaseous protein ions varied as a function of time following IR activation, indicating that noncovalent interactions can be transiently disrupted but are also capable of reforming as the ions relax into alternative, electrostatically stabilized conformations. Although these investigations focused on the folding kinetics of monomers, they provide a general conceptual framework for understanding how gas-phase protein ions undergo internal reorganization. The importance of ion pairs and their rearrangement was brought in the forefront by Loo and Loo, who proposed an alternative model for asymmetric charge partitioning in MPCs. In this mechanistic view, the heterolytic cleavage of intersubunit salt bridges inherently distributes charge asymmetrically between dissociation products, and such charge partitioning does not require (extensive) unfolding.^80^ IM-MS studies have shown that even under asymmetric charge partitioning, ejected monomers can remain remarkably compact, exhibiting only modest increases in CCS despite acquiring several-fold more charge.^81,82^ Asymmetric dissociation events, therefore, may generate monomeric ions that are only partially unfolded rather than fully solvent accessible. This limited unfolding is consistent with the differences observed in fragmentation maps obtained by CxD and nTD, particularly for ADH (Figure 6) and PK (Figure S16). It is tempting to attribute the complementarity between these two approaches (at least partly) to the heterolytic scission of intersubunit ion pairs, whereby the residues previously engaged in salt bridges become liberated in the highly charged expelled monomers, increasing their accessibility. However, this mechanism alone is unlikely to account for the reduced fragmentation observed by CxD in regions that are readily sequenced by nTD. PK provides an illustrative example: both the N- and C-termini of PK have been reported to participate in subunit interaction interfaces.^19^ The fact that both termini are engaged in noncovalent interactions is supported by ETD results, which show very few product ions, with sequence coverages reaching only 4.9% and 1.3% with and without supplemental HCD activation, respectively. IR irradiation concurrent to the ETD reaction could unfold the tetramer such that a coverage of 27.8% was attained. As shown in Figure S16, nTD yields extensive cleavage toward the N-terminus, whereas CxD predominantly generates product ions explaining the C-terminal region. The simultaneous increase in accessibility of the interfacial C-terminus and attenuated fragmentation in previously accessible regions suggests structural rearrangement of the expelled monomer, in which newly formed electrostatic interactions selectively constrain portions of the polypeptide chain and thereby modulate fragmentation behavior. In addition to the mechanistic considerations underlying the comparable sequence coverages attained in nTD and CxD MS experiments, it is important to note the practical limitations associated with the latter approach. CxD MS involves the release of monomer subunits—a step that can substantially reduce signal intensity due to (i) incomplete monomer ejection and (ii) an additional stage of precursor isolation (in the case of true MS^3^). In our experiments, both the isolated precursor ions and the resulting fragment mass spectra exhibited signal intensities that were at least an order of a magnitude lower than those obtained in nTD MS (as seen in Figure 1). Consequently, the average S/N of fragments in MS^3^ spectra was considerably poorer, likely precluding the observation of low-abundance product ions.

## Conclusions

Overall, this work demonstrated the potential and versatility of the Tribrid Orbitrap platform for native top-down MS, and how the addition of an IR laser can significantly expand the tool set available for the investigation of MPCs in the near future. We believe the results presented here exemplify how IR irradiation can be applied for native MS utilized as either a structural biology tool (i.e., nTD MS) or to complement denaturing top-down MS experiments by interrogating unknown MPCs in large-scale studies (i.e., CxD MS). The use of low-energy IR photons offers improvements in MPC desolvation, subunit ejection, and subunit sequencing. More specifically, we speculate that IRMPD could efficiently be employed for untargeted experiments aimed at characterizing MPC mixtures separated via either liquid chromatography^71,83^ or capillary electrophoresis,^84^ while AI-ETD showed greater potential for achieving extensive sequence coverage of large MPC subunits. However, additional studies must be conducted to further evaluate fundamental differences in HCD and IRMPD fragmentation propensities, as well as to mechanistically explain the observed differences (specifically, regarding the relative abundance of *b*/*y* versus *c*/*z*-ions) in AI-ETD fragmentation of subunits as part of an MPC or after their ejection. Future works will have to address fragmentation products that could not be annotated in ETD, EThcD and AI-ETD mass spectra, which (particularly when supplemental activation is used) may represent internal fragments useful for further extending sequence coverage.^85^ Furthermore, including UVPD may offer complementary insights both in sequence coverage and ligand- or interface-bound regions by accessing higher-energy fragmentation pathways.^39,47,86^

## Supplementary Material



## Notes

Supporting figures and tables including observed masses for each complex and suggestion for MPC composition; summary sequence coverage results for all proteins; comparison of ETD, EThcD and AI-ETD for the sequencing of ADH; off-line mass recalibration of product ion mass spectra in TDValidator; visual comparison of different ion activation techniques for the gas-phase sequencing of enolase and PK monomers; confirmation of the V58T amino acid substitution in ADH; fragments observed for an ADH monomer proteoform lacking NtAc; comparison of IRD and sCID for desolvation; ADH monomer ejection using sCID, HCD and IRMPD; comparison of AI-ETD, HCD and IRMPD for the sequencing of enolase at the CxD and nTD MS levels; comparison of AI-ETD, HCD and IRMPD for the sequencing of PK at the CxD and nTD MS levels; PIA plots on the HCD and IRMPD of enolase; PIA plots on the HCD and IRMPD of PK; product ions confirming the presence of bound Zn (II) in ADH; PIA plot on the AI-ETD of enolase; PIA plot on the AI-ETD of PK; combined fragmentation maps for enolase and for PK.(PDF)
