# Supplementary material for: On the Utility of Infrared Photoactivation for Native Top-Down and Complex-Down Orbitrap Mass Spectrometry of Soluble Proteoform Complexes
Source: J Am Soc Mass Spectrom. 2026 Feb 11;37(3):682–94. doi: 10.1021/jasms.5c00385 (PMC12964546; doi:10.1021/jasms.5c00385)
Supplement: Supplementary file 1 [file js5c00385_si_001.pdf]

SUPPORTING INFORMATION

On the utility of infrared photoactivation for native top-down and complex-down Orbitrap mass spectrometry of soluble proteoform complexes

Cynthia Nagy,[1] Linda B. Lieu,[2] Christopher Mullen,[3] Graeme C. McAlister,[3] Rafael D. Melani,[3] Joshua D. Hinkle,[3] Luca Fornelli[1,2*]

[1] *School of Biological Sciences, University of Oklahoma, Norman, OK, 73019 USA*

[2] *Department of Chemistry and Biochemistry, University of Oklahoma, Norman, OK, 73019 USA*

[3] *Thermo Fisher Scientific, San Jose, CA, 95134 USA*

*To whom correspondence should be addressed:

Luca Fornelli, School of Biological Sciences, Richards Hall 411B, 730 Van Vleet Oval, Norman, OK, 73019. Phone: 405-325-1483; Fax: 405-325-6202; Email: luca.fornelli@ou.edu

Keywords: complex-down, native top-down, Orbitrap, multiproteoform complexes, photoactivation, electron transfer dissociation

Contents

Table S1 3
Table S2 4
Table S3 5
Figure S1 6
Figure S2 7
Figure S3 8
Figure S4 9
Figure S5 10
Figure S6 11
Figure S7 12
Figure S8 13
Figure S9 14
Figure S10 15
Figure S11 16
Figure S12 17
Figure S13 18
Figure S14 19
Figure S15 20
Figure S16 21

Table S1

Observed masses for each complex and suggestion for MPC composition. Intact masses were obtained with IRD in the case of enolase and ADH, for PK sCID was used. Variations are indicated relative to the sequence observed in UniProt.

UniProt accession #	Observed mass (Da)	MPC composition	Theoretical mass (Da)	Mass error (ppm)
Enolase				
P00924	93,342.39	2 × (MetOFF; I242V)	93,341.84	5.89
PK				
P11974	231,787.72	3 × (MetOFF; NtAc; S400A)<br>1 × (MetOFF; NtAc)	231,787.40	1.38
	231,862.82	3 × (MetOFF; NtAc; S400A)<br>1 × (MetOFF; NtAc)<br>1 × BME covalent mod.	231,863.4	-2.50
	229,573.95	2 × (MetOFF; NtAc; S400A)<br>1 × (MetOFF; NtAc)<br>1 × (trunc [23-531])	229,572.22	7.54
ADH				
P00330	147,499.08	4 × (MetOFF; NtAc; V58T; I152V)<br>8 × Zn^{2+}	147,499.12	-0.27

Table S2

Summary sequence coverage results for all proteins: alcohol dehydrogenase (ADH), enolase, and pyruvate kinase (PK). Best results are reported from the respective fragmentation techniques for CxD MS and nTD MS experiments.

	Activation		Sequence coverage (%)	
Fragmentation Technique	CxD	nTD	CxD	nTD
ADH				
AI-ETD	~4.5 W; 15 ms ETD	~2.7 W; 10 ms ETD	16.2	35.5
HCD	NCE 60%	NCE 50%	35.0	37.9
IRMPD	~9 W	~6.9 W	27.5	33.8
Enolase				
AI-ETD	~3.9 W; 7 ms ETD	~2.7 W; 7 ms ETD	12.9	21.6
HCD	NCE 50%	NCE 50%	16.3	31.3
IRMPD	~5.7 W	~6.3 W	12.2	20.9
PK				
AI-ETD	~3.3 W; 15 ms ETD	~2.7 W; 20 ms ETD	23.3	27.8
HCD	NCE 60%	NCE 60%	13.8	13.0
IRMPD	~8.1 W	~6 W	12.1	10.6

Table S3

Comparison of results obtained via ETD, EThcD, and AI-ETD for the sequencing of ADH, including sequence coverage and relative frequency of b/y-type vs c/z-type ions.

	CxD MS			nTD MS		
Fragmentation Technique	Sequence coverage (%)	Relative b/y (%)	Relative c/z (%)	Sequence coverage (%)	Relative b/y (%)	Relative c/z (%)
ETD	4	13.3	86.7	11.8	0	100
EThcD	12.4	14.9	85.1	14.2	20.7	79.3
AI-ETD	16.2	41.4	58.6	35.5	39.6	60.4

Figure S1

The off-line recalibration of product ions was performed using the built-in calibration function in TDValidator. This feature applies a uniform mass shift to the selected ion population so that the observed *m/z* values are centered more closely around 0 ppm error relative to theoretical values. Applying this functionality improves the alignment between observed and theoretical matches during manual validation. An example workflow is shown below.

The Experimental Data tab contains the calibration function used for off-line calibration. TDValidator visualizes the overall ppm error distribution for the matched product ions (Figure S1-A). In the example below, the average mass error was +3.54 ppm. To center the observed ion population around 0 ppm, we applied a calibration shift of –3.5 ppm. This recalibrated distribution is shown in the screenshot below, where the average mass error is now +0.21 (Figure S1-B)

A

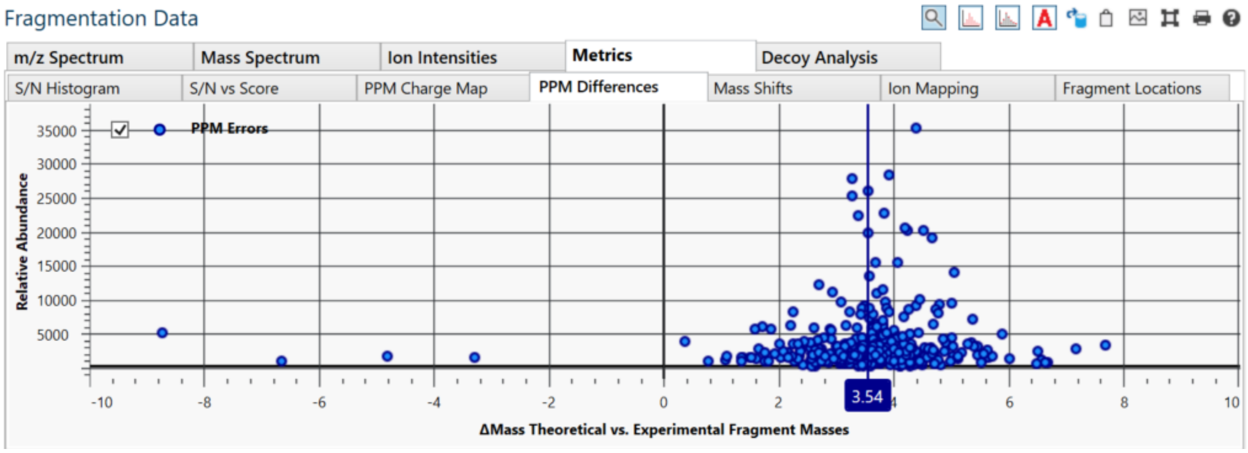

B

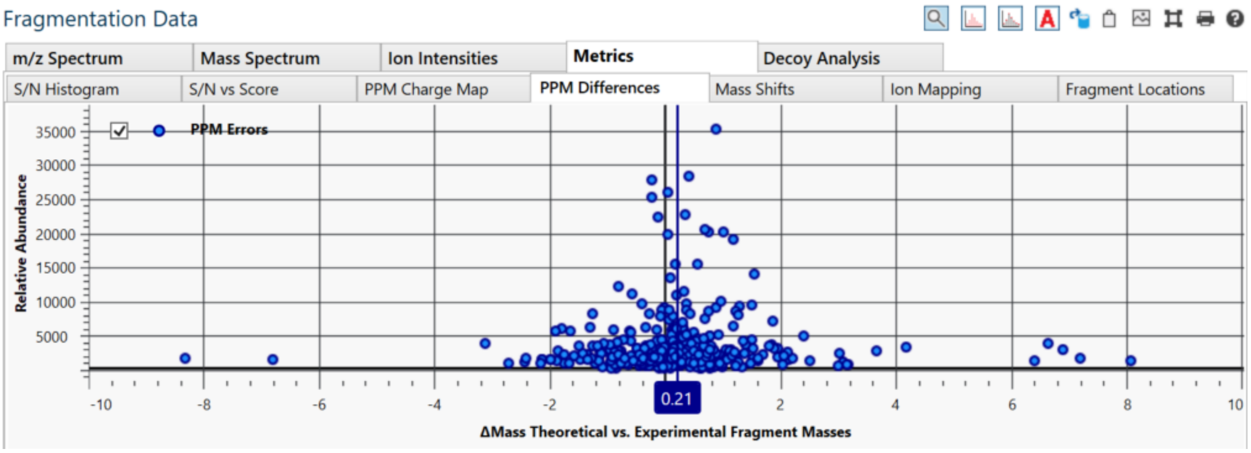

Figure S1. Off-line mass recalibration of product ion mass spectra in TDValidator. (A) Before recalibration and (B) after recalibration.

Figure S2

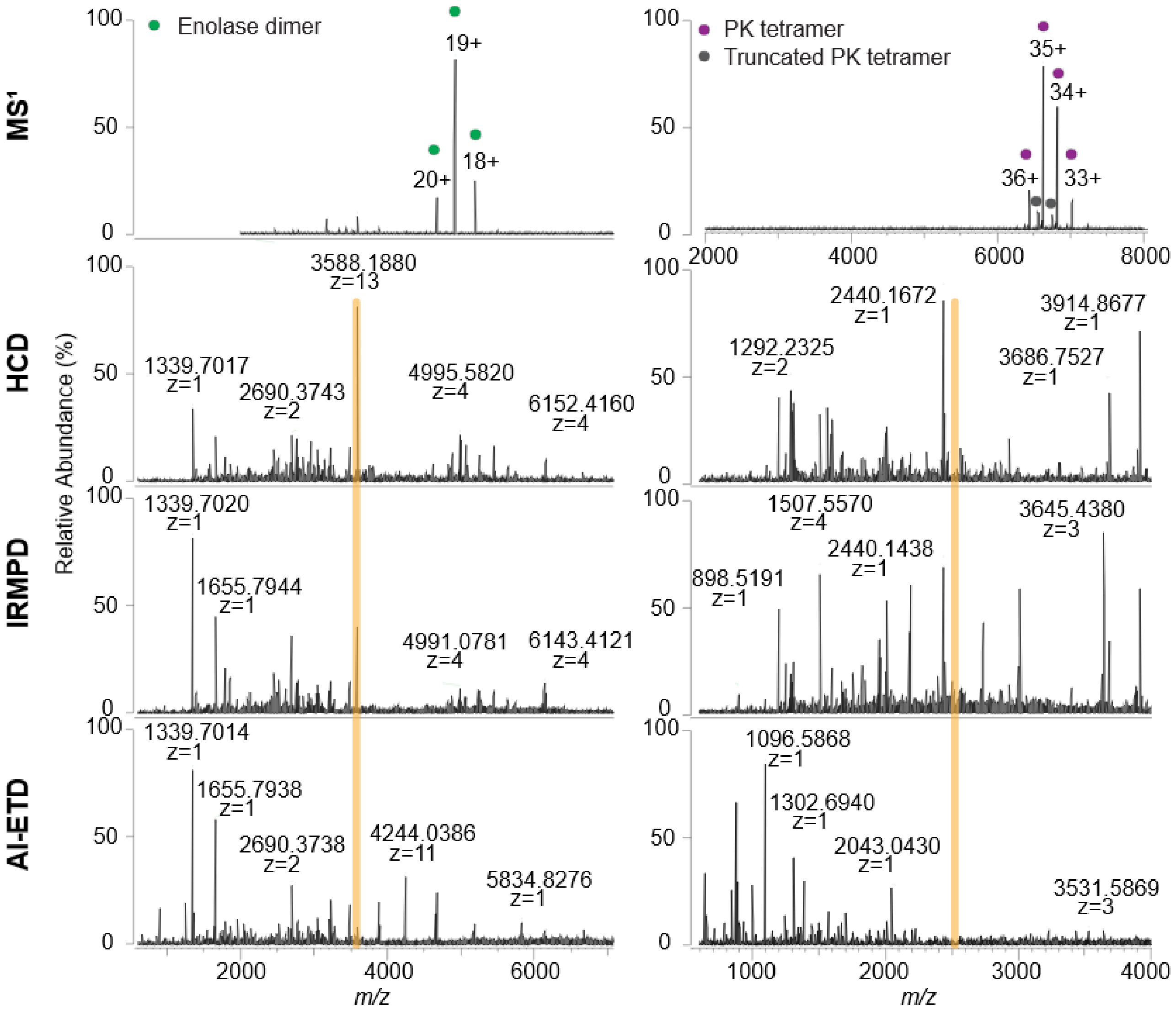

Figure S2. Comparison of different ion activation techniques for the gas-phase sequencing of ejected monomers. Left panel: enolase; right panel: PK. Top panel shows intact mass spectra of the two complexes; consecutive panels in vertical order display HCD, IRMPD and AI-ETD spectra. Precursor regions are highlighted in orange.

Figure S3

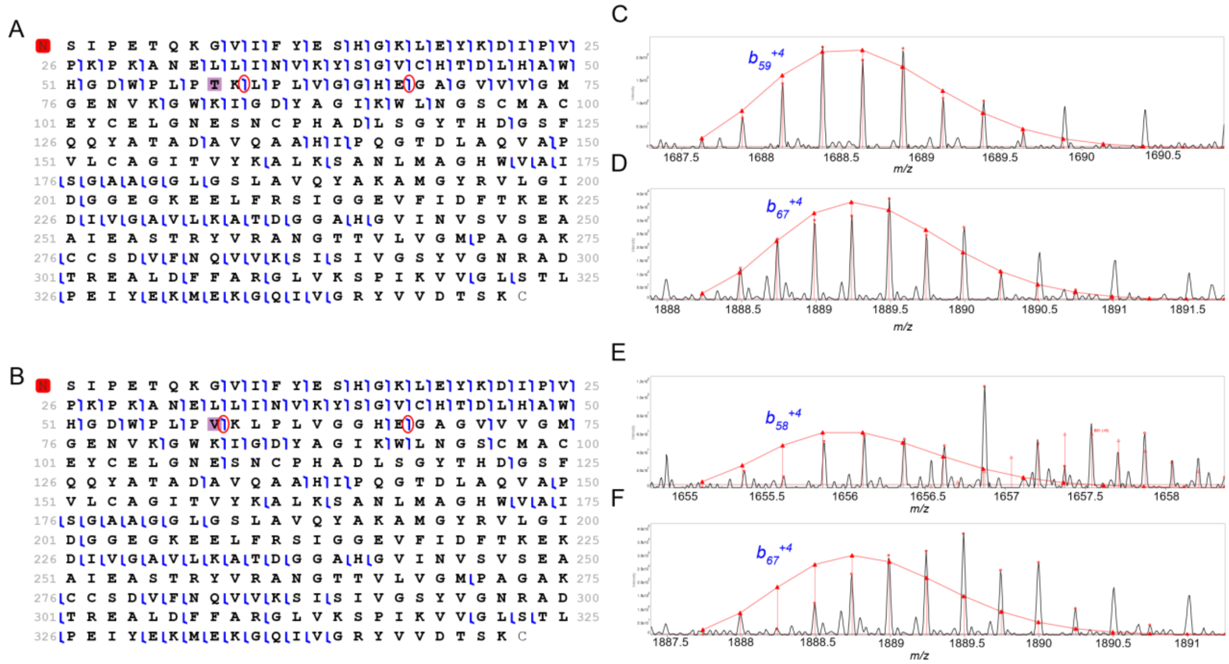

Figure S3. Comparison between ADH HCD monomer fragmentation maps accounting for the V58T amino acid substitution. (A) T58 and (B) V58 variants. Product ions within the 58-139 sequence region are compared, where (C) b_{59}^{4+} is unique to the T58 variant and (E) b_{58}^{+4} is unique to the V58 variant. b_{67}^{+4} is matched in both (D) T58 and (F) V58 variants.

Manually validated fragmentation maps of the (A) T substitution and (B) original V residue in position 58 show substantial discrepancies in the region spanning positions 58-139. All C-terminal product ions as well as N-terminal fragments upstream of position 58 have been manually validated with high confidence. In the case of T58 variant, several fragments matched with very good fit scores that are not even automatically assigned in the V58 variant, such as product ion b_{59}^{4+} shown in part (C). While we do observe a product ion that is assigned in the V58 variant and not in the substituted T58 (E), the poor ion statistics would make it likely to be removed during our manual validation process. Additionally, most of the product ions that appear in the case of T58 show poorer isotopic fitting with V at position 58. This is exemplified in parts (D) and (F), which illustrate the same fragment (b_{67}^{+4}) matched for the T58 and V58 variants, respectively, but with varying fit – the skewed isotopic fit in the case of V58 would have us exclude this match during manual validation.

Figure S4

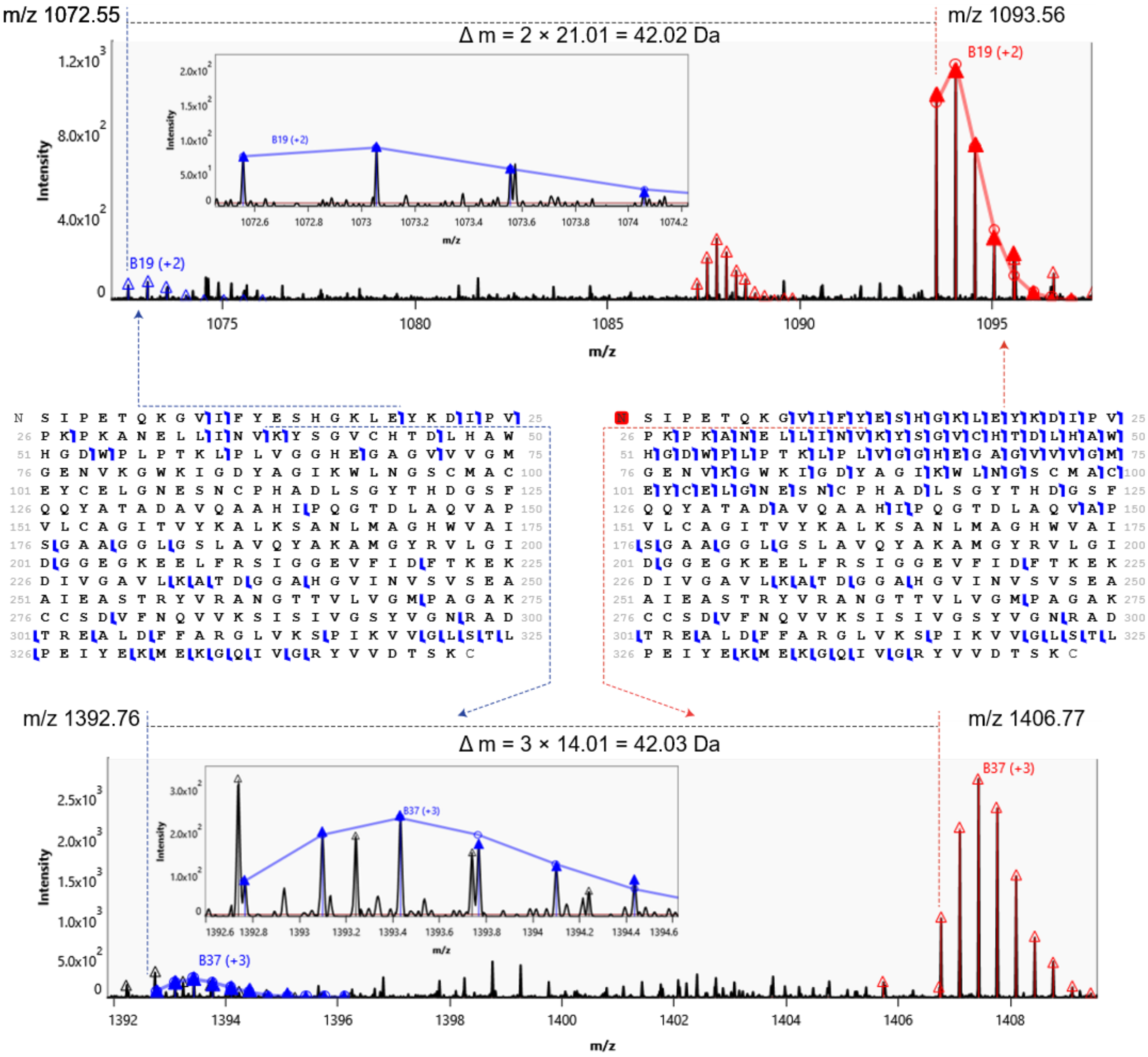

Figure S4. IRMPD nTD MS spectrum of ADH tetramer, confirming the presence of an extra ADH monomer proteoform, which contains no N-terminal acetylation (NtAc). Center: fragmentation maps for the proteoforms differing only in NtAc. Top and bottom: examples of -42 Da mass shifts observed in fragment mass spectra, where isotopic distributions highlighted in blue correspond to the proteoform lacking NtAc, and the color red indicates the proteoform containing NtAc.

Figure S5

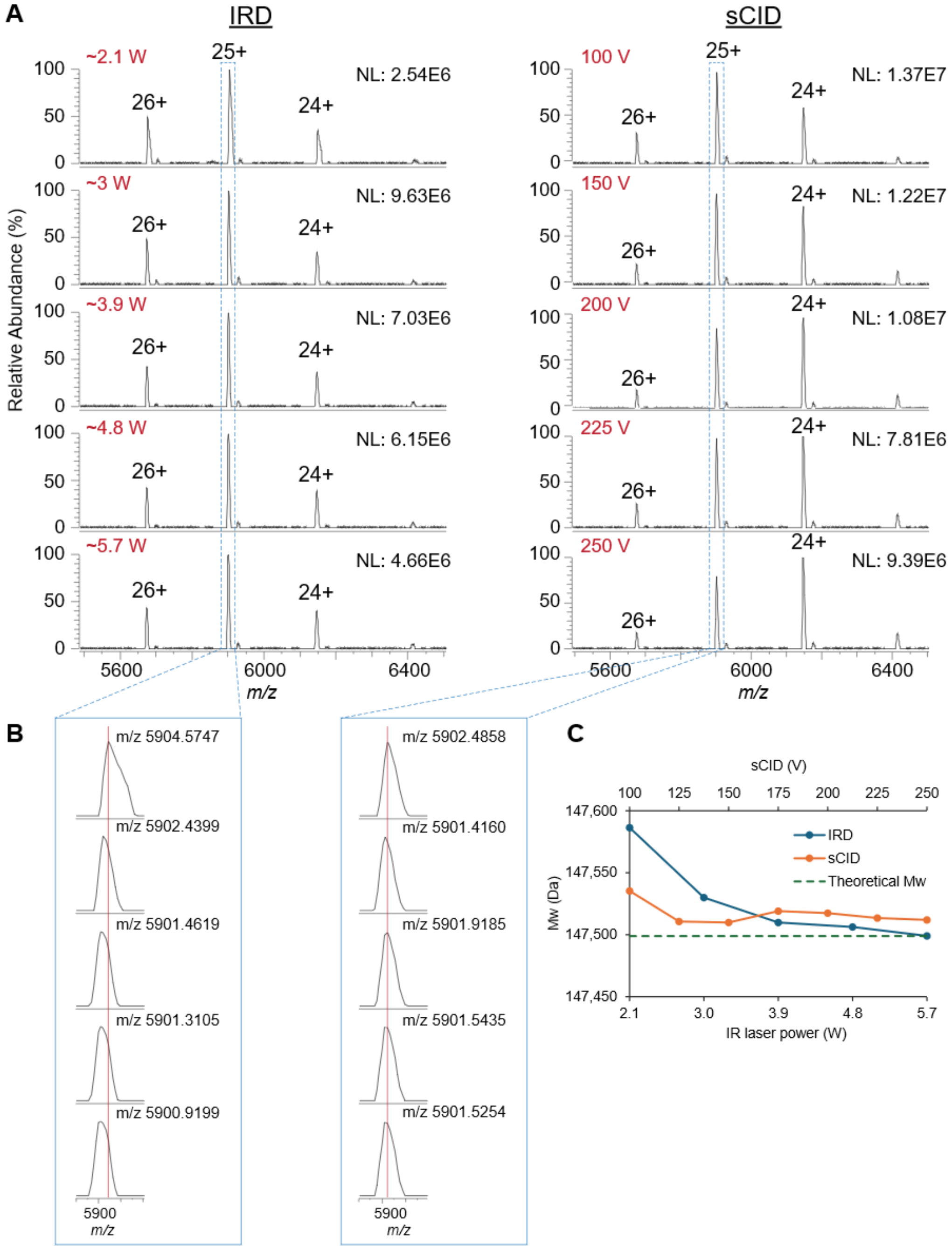

Figure S5. Comparison of IRD and sCID for desolvation. (A) Intact mass spectra for ADH tetramer using IRD and sCID with corresponding laser powers and voltages used. (B) Zoomed-in sections of the 25+ charge state showing the change in peak shape. Red lines indicate initial positions of peak apex (~2.1 W laser power and 100 V for IRD and sCID, respectively). (C) Average molecular weights (Mw) obtained using various laser powers (for IRD) and voltages (for sCID). The theoretical Mw for ADH homotetramer with 8 Zn^{2+} ions bound is ~147,499 Da.

Figure S6

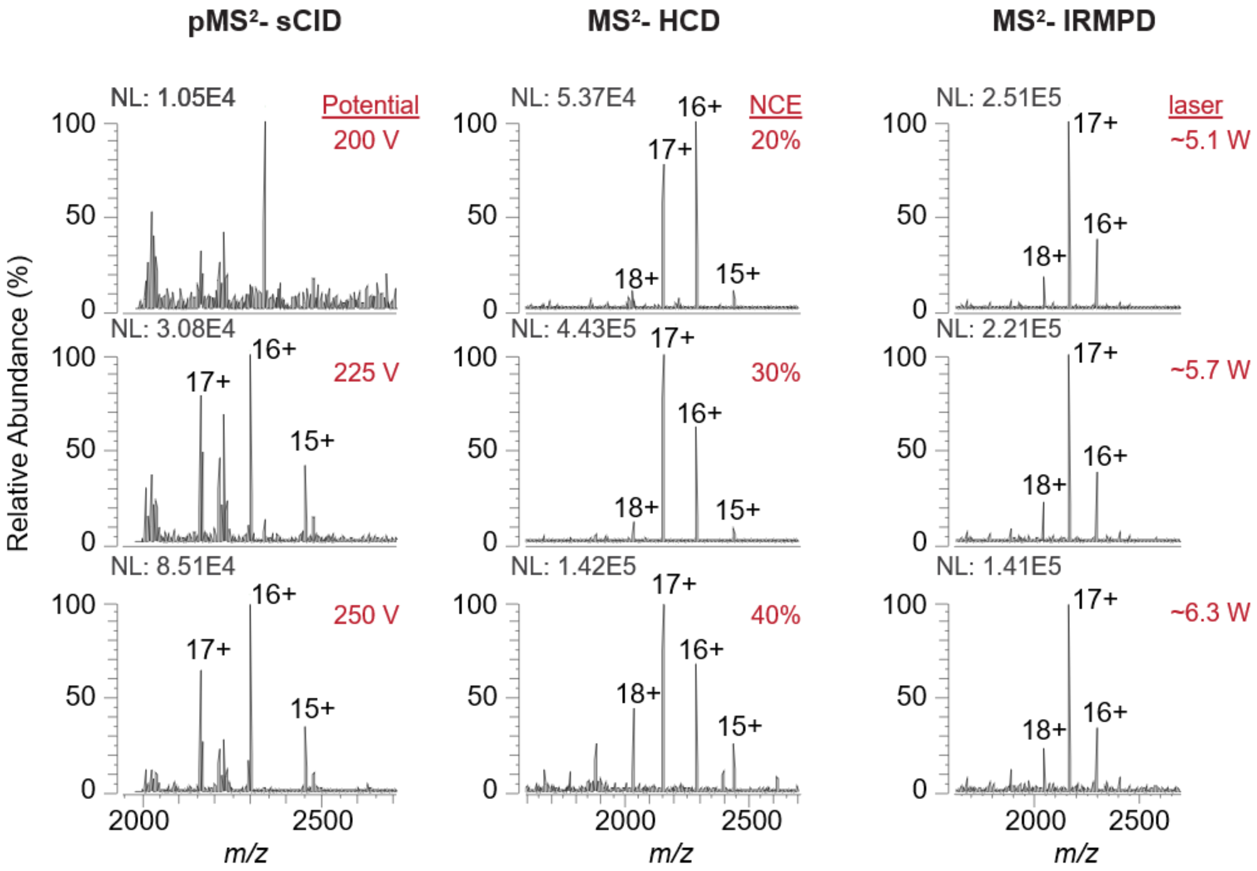

Figure S6. ADH monomer ejection using sCID, HCD and IRMPD.

Figure S7

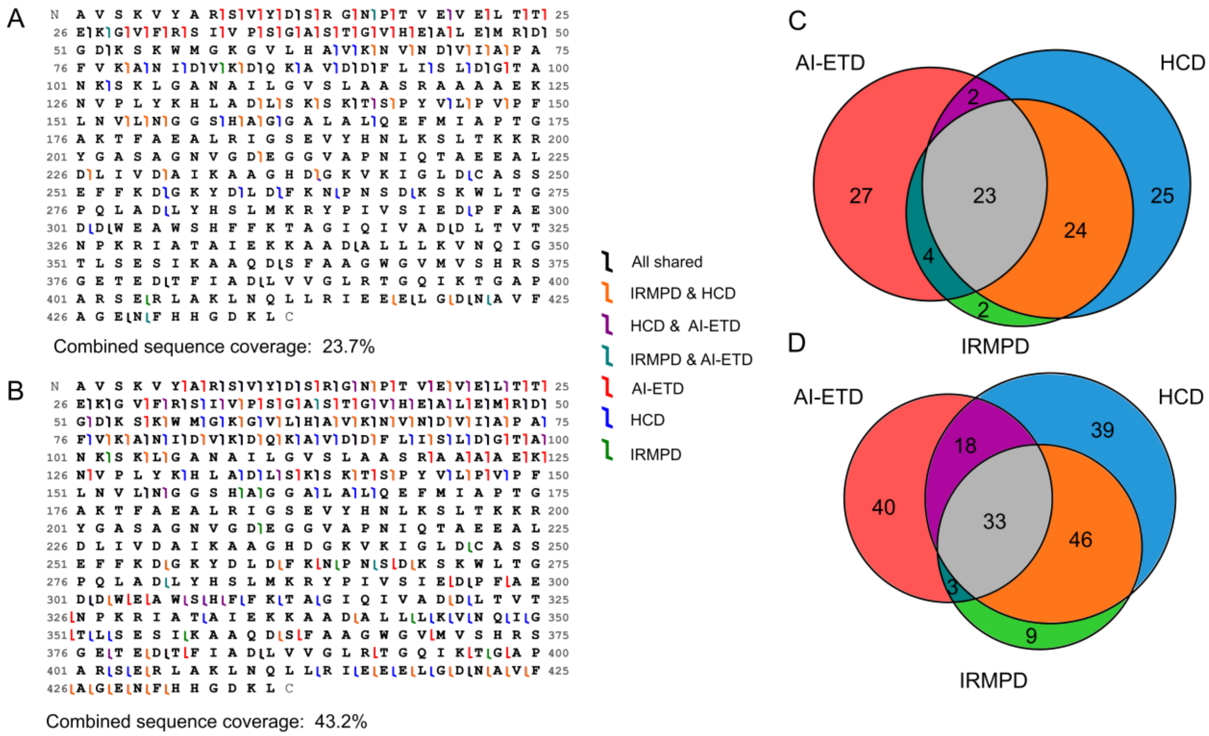

Figure S7. Comparison of unique product ions in AI-ETD, HCD, and IRMPD experiments for enolase at the CxD MS and nTD MS levels. Fragmentation maps obtained in (A) CxD MS and (B) nTD MS experiments. Venn diagrams display the distribution of unique product ions at the (C) CxD MS and (D) nTD MS levels. Given the different types of product ions produced by AI-ETD and IRMPD/HCD, Venn diagrams are based on backbone cleavage regardless of the nature of matched fragment.

Figure S8

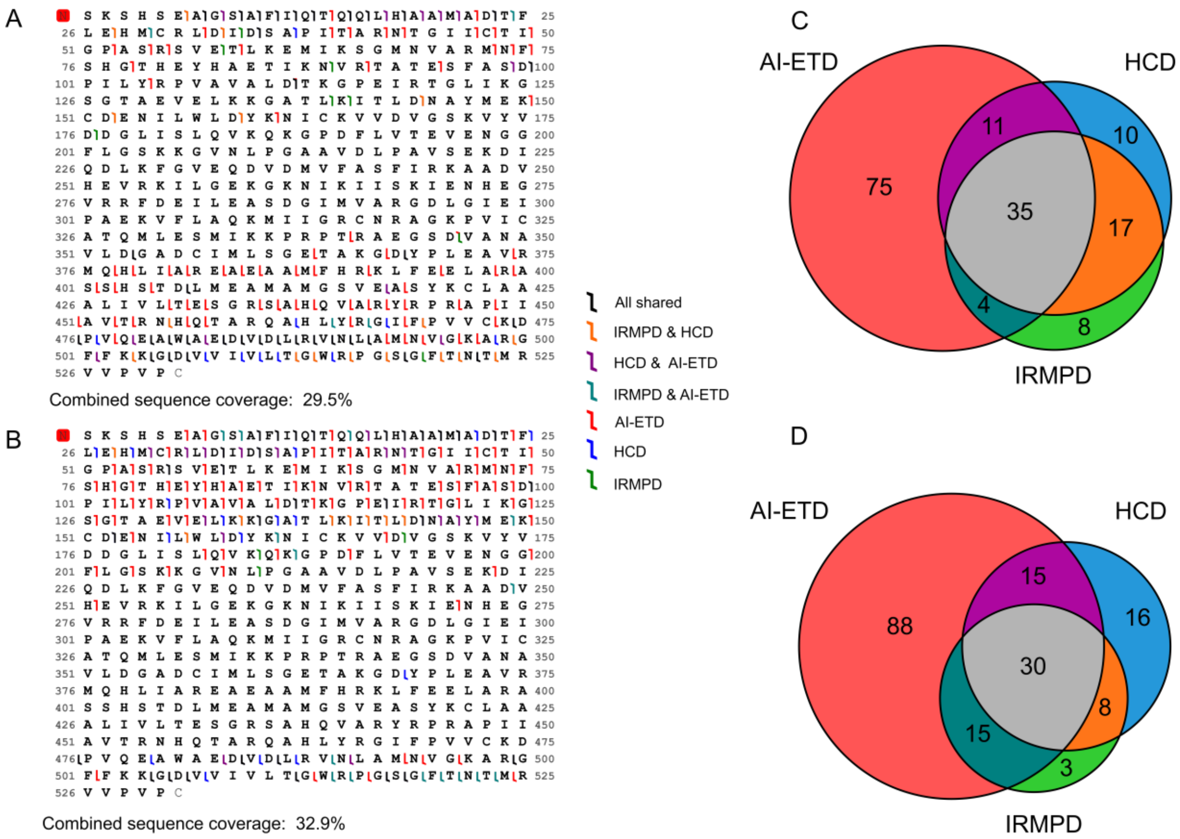

Figure S8. Comparison of unique product ions in AI-ETD, HCD, and IRMPD experiments for PK at the CxD MS and nTD MS levels. Fragmentation maps obtained in (A) CxD MS and (B) nTD MS experiments. Venn diagrams display the distribution of unique product ions at the (C) CxD MS and (D) nTD MS levels. Given the different types of product ions produced by AI-ETD and IRMPD/HCD, Venn diagrams are based on backbone cleavage regardless of the nature of matched fragment.

Figure S9

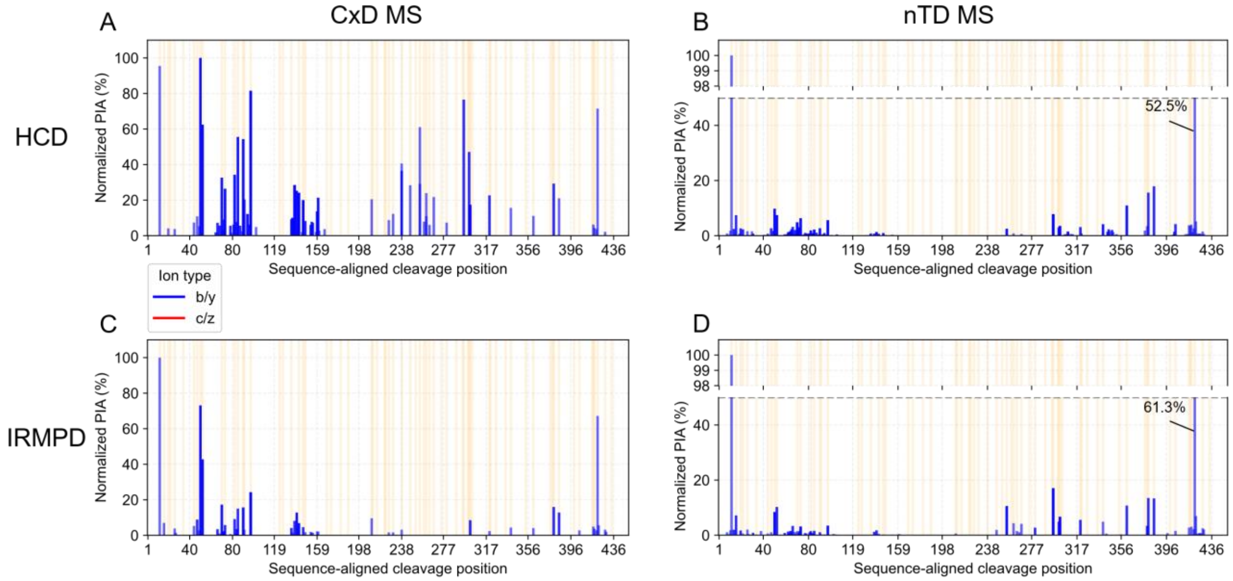

Figure S9. Results of product ion abundance (PIA) analysis for HCD and IRMPD experiments on enolase, comparing CxD MS and nTD MS. (A) HCD CxD MS, (B) HCD nTD MS, (C) IRMPD CxD MS, and (D) IRMPD nTD MS. Regions highlighted in yellow indicate D/*, E/*, and */P cleavage sites.

Figure S10

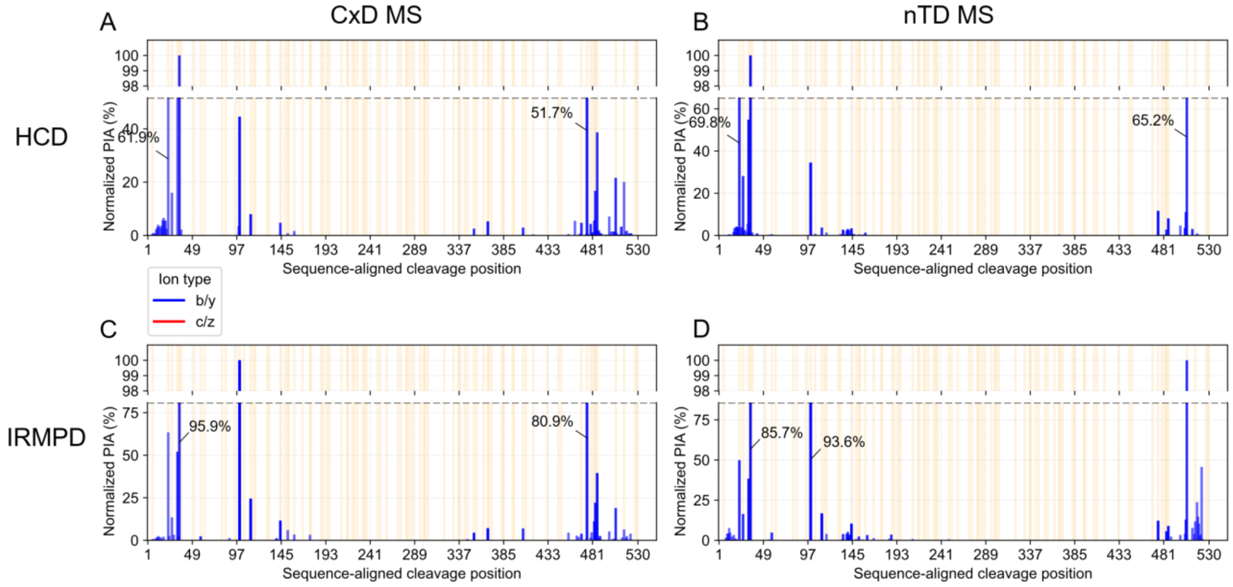

Figure S10. Results of product ion abundance (PIA) analysis for HCD and IRMPD experiments on PK, comparing CxD MS and nTD MS. (A) HCD CxD MS, (B) HCD nTD MS, (C) IRMPD CxD MS, and (D) IRMPD nTD MS. Regions highlighted in yellow indicate D/*, E/*, and */P cleavage sites.

Figure S11

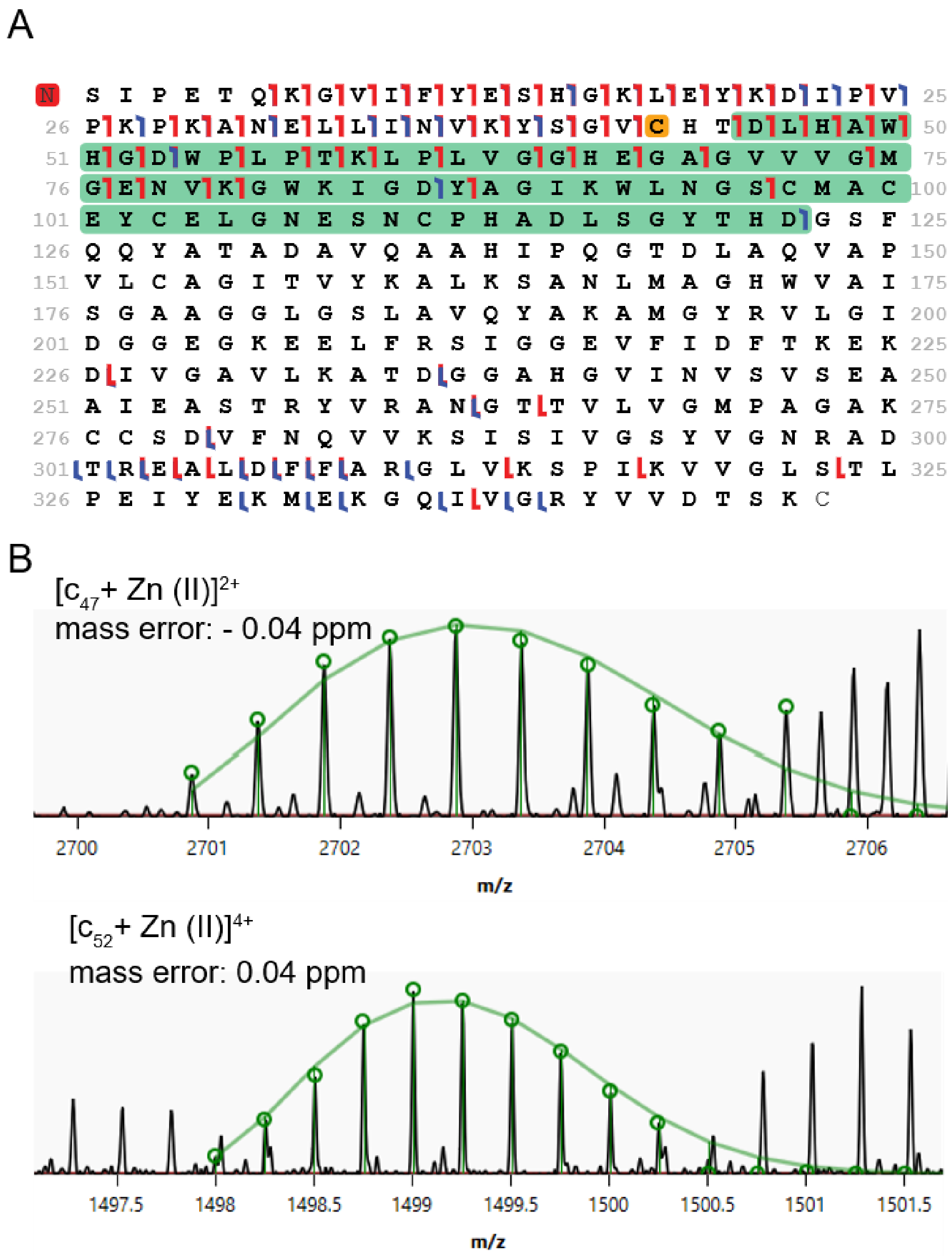

Figure S11: Confirming the localization of Zn (II) binding in ADH. (A) Fragmentation map obtained by activating ADH tetramer with AI-ETD. Cys 43 residue in yellow indicates bound Zn (II). Sequence region highlighted in green contains fragments confirming the mass shift indicative of Zn (II) -binding. (B) Two examples of matched fragments containing the Zn (II).

Figure S12

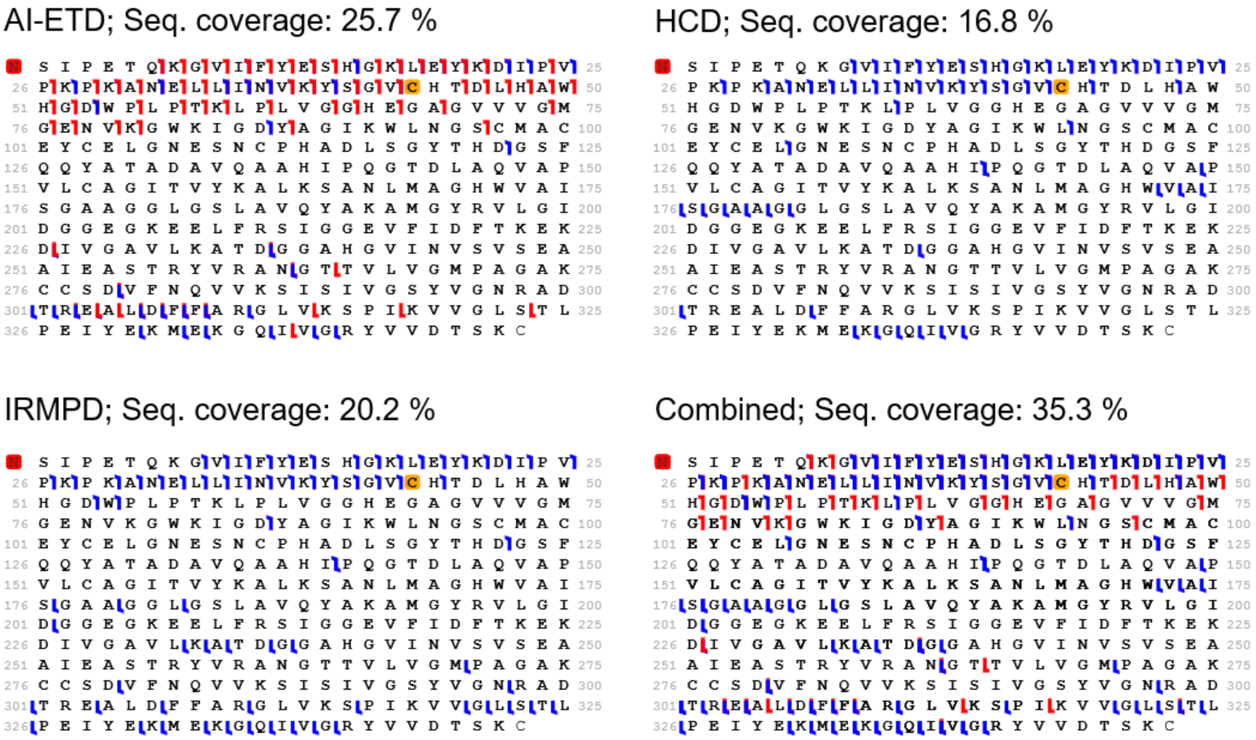

Figure S12: Individual and combined fragmentation maps indicating the cleavage sites giving rise to product ions with bound Zn (II). Sequence coverages for the proteoform carrying the Zn (II) at Cys43 are also indicated.

Figure S13

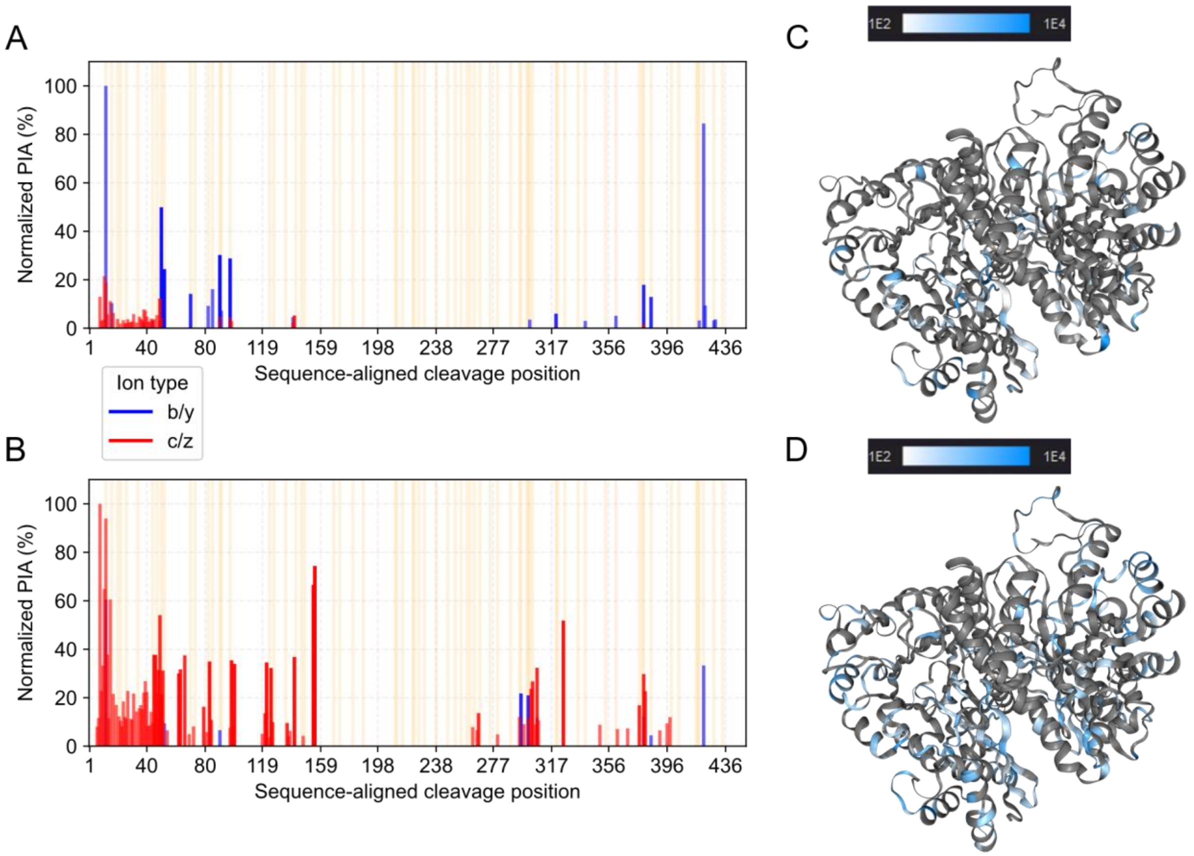

Figure S13. Product ion abundance (PIA) plots for the AI-ETD fragmentation of the enolase. (A) CxD MS and (B) nTD MS experiments. Regions highlighted in yellow indicate D/*, E/*, and */P cleavage sites, while *b*/*y*-ions are displayed in blue and c/z-ions in red. (C) and (D) show the crystal structure of enolase with assigned product ions from CxD and nTD MS experiments, respectively. Matched backbone cleavages are colored in shades of blue as a function of their intensity.

Figure S14

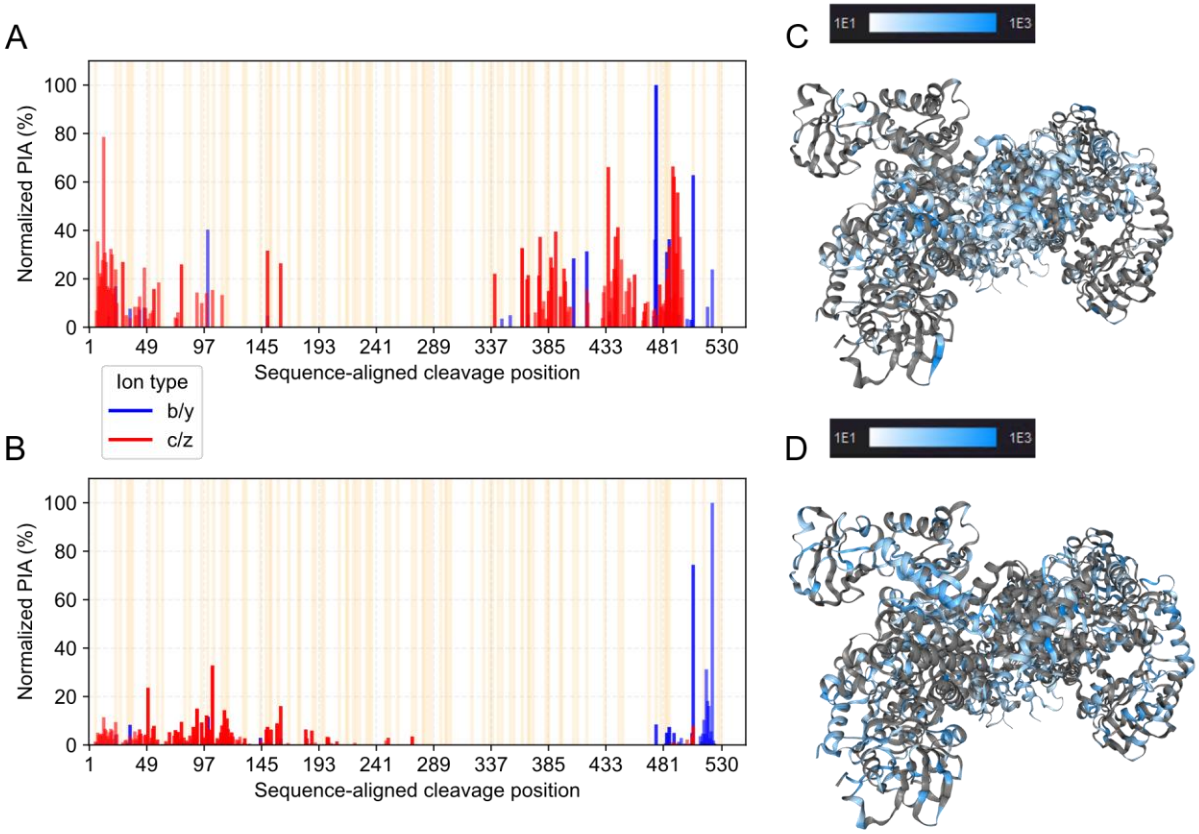

Figure S14. Product ion abundance (PIA) plots for the AI-ETD fragmentation of the pyruvate kinase. (A) CxD MS and (B) nTD MS experiments. Regions highlighted in yellow indicate D/*, E/*, and */P cleavage sites, while *b*/*y*-ions are displayed in blue and c/z-ions in red. (C) and (D) show the crystal structure of PK with assigned product ions from CxD and nTD MS experiments, respectively. Matched backbone cleavages are colored in shades of blue as a function of their intensity.

Figure S15

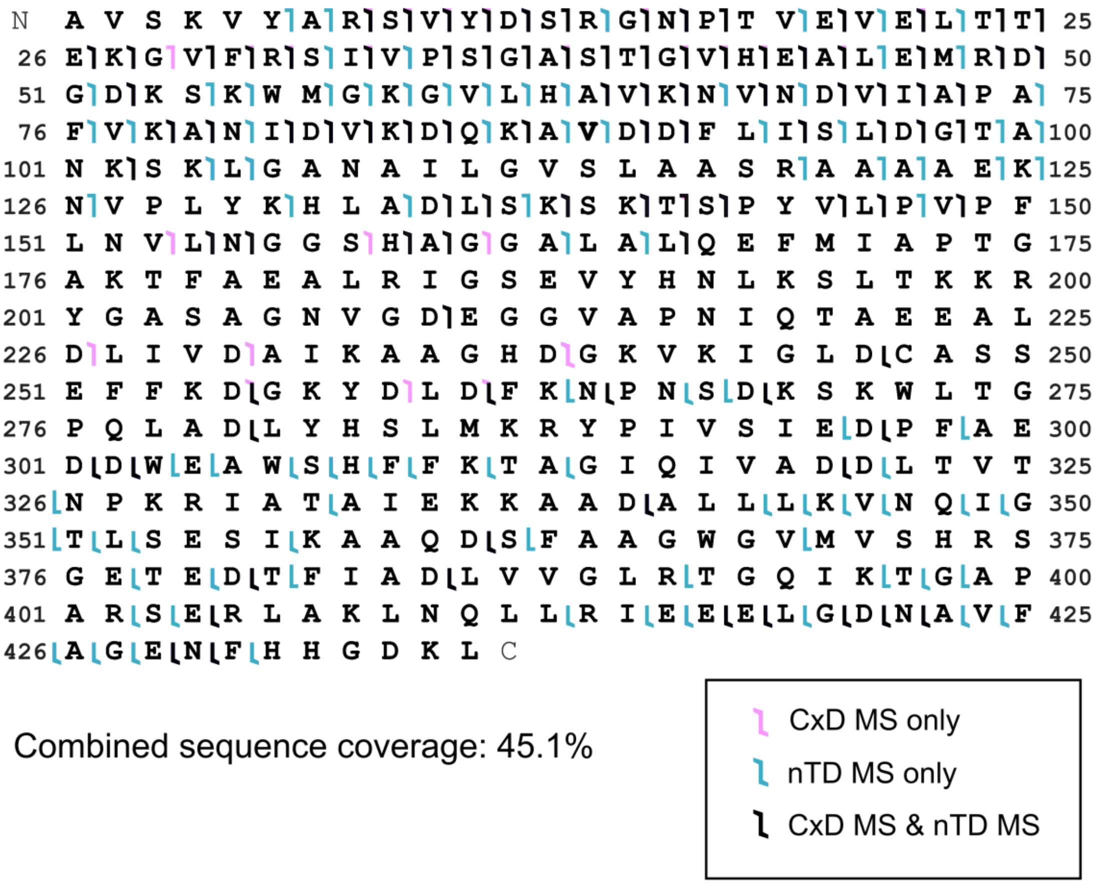

Figure S15. Combined fragment map showing results from AI-ETD, HCD, and IRMPD experiments for enolase analyzed via CxD MS and nTD MS. Pink brackets indicate fragments detected only in CxD MS experiments, blue brackets indicate fragments detected only in nTD MS experiments, and black brackets indicate fragments detected in both. CxD experiments: HCD NCE 50%, IRMPD laser power ~5.7 W, AI-ETD with ~3.9 W laser power and 7 ms ETD reaction time; nTD experiments: HCD NCE 50%, IRMPD laser power ~6.3 W, AI-ETD with ~2.7 W laser power and 7 ms ETD reaction time.

Figure S16

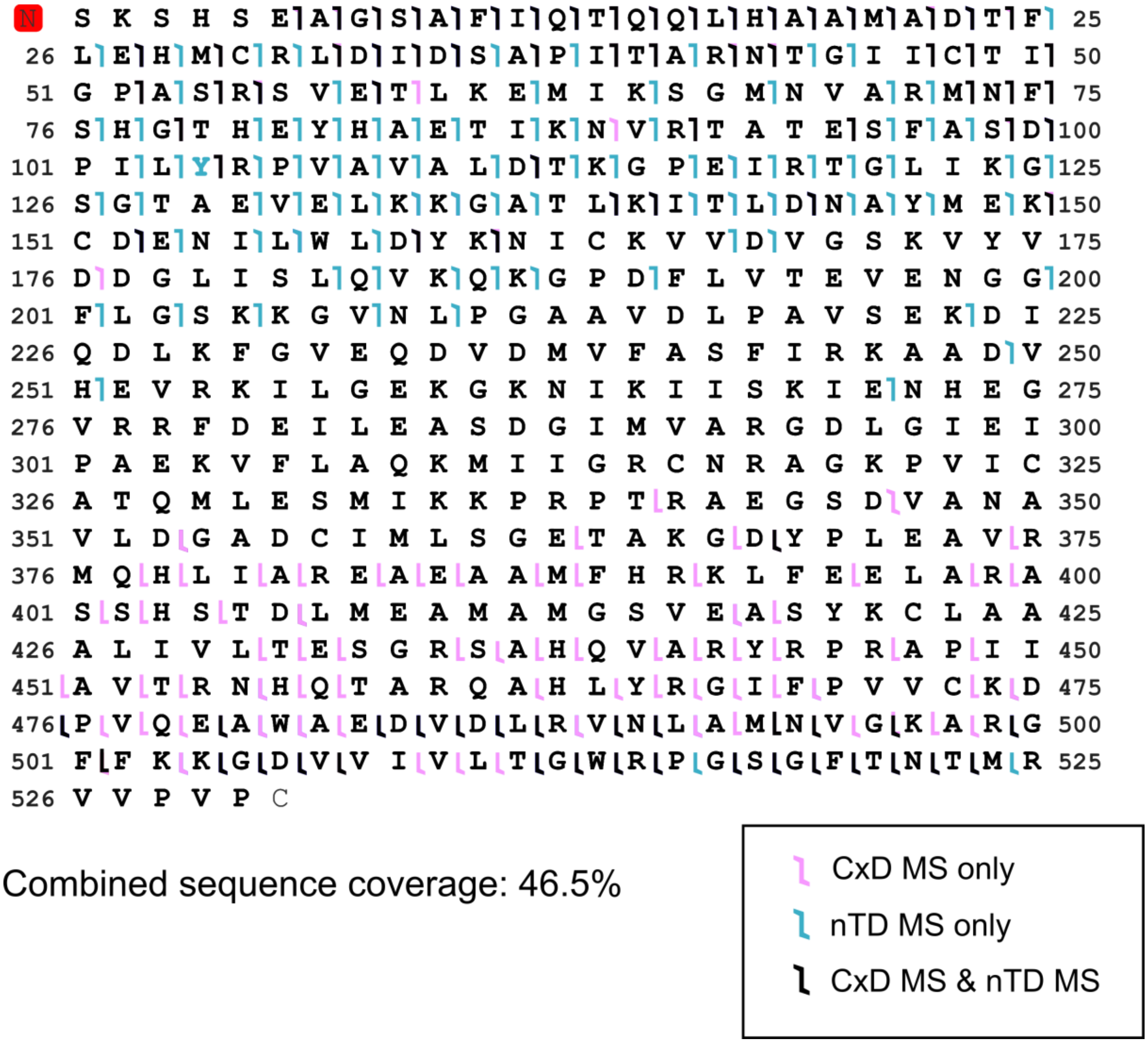

Figure S16. Combined fragment map showing results from AI-ETD, HCD, and IRMPD experiments for PK analyzed via CxD MS and nTD MS. Pink brackets indicate fragments detected only in CxD MS experiments, blue brackets indicate fragments detected only in nTD MS experiments, and black brackets indicate fragments detected in both. CxD experiments: HCD NCE 60%, IRMPD laser power ~8.1 W, AI-ETD with ~3.3 W laser power and 15 ms ETD reaction time; nTD experiments: HCD NCE 60%, IRMPD laser power ~6 W, AI-ETD with ~2.7 W laser power and 20 ms ETD reaction time.
